# Complex 3D bioprinting methods

**DOI:** 10.1063/5.0034901

**Published:** 2021-03-11

**Authors:** Shen Ji, Murat Guvendiren

**Affiliations:** 1 Otto H. York Chemical and Materials Engineering, New Jersey Institute of Technology, 161 Warren Street, 150 Tiernan Hall, Newark, New Jersey 07102, USA; 2 Department of Biomedical Engineering, New Jersey Institute of Technology, University Heights, Newark, New Jersey 07102, USA

## Abstract

3D bioprinting technology is evolving in complexity to enable human-scale, high-resolution, and multi-cellular constructs to better mimic the native tissue microenvironment. The ultimate goal is to achieve necessary complexity in the bioprinting process to biomanufacture fully-functional tissues and organs to address organ shortage and lack of patient-specific disease models. In this Review, we presented an in-depth overview of complex 3D bioprinting approaches including evolution of complex bioprinting, from simple gel-casting approach to multi-material bioprinting to omnidirectional bioprinting approaches, and emerging bioprinting approaches, including 4D bioprinting and *in situ* bioprinting technologies.

## INTRODUCTION

Three-dimensional (3D) bioprinting is an additive manufacturing technology that enables biomanufacturing of living tissues from a 3D digital image.^1^ The promise of bioprinting is the creation of human-scale patient-specific tissues/organs that are anatomically and physiologically similar to the patient's native tissue by using patient's own medical images and cells.^2^ This technology already enabled fabrication of small-scale tissues,^3–5^ and in the short-term, these bioprinted tissues could potentially address the lack of functional *in vitro* tissue/disease models for personalized medicine and drug screening. In the long-term, bioprinting shows strong potential to address the shortage of implantable organs.^6,7^ To achieve these short- and long-term goals, it is crucial to capture the architectural, structural, mechanical, and biochemical complexity of the native tissue. This requires the bioprinting process to evolve from small-scale, low-resolution, single or dual cell and biomaterial printing to human-scale, high-resolution, multi-cellular, and multi-biomaterial printing.^8–10^

3D bioprinting refers to printing of live cells, and a bioink is defined as a bioprintable formulation, which is composed of live cells alone (cell-based bioinks) or combined with a hydrogel formulation (hydrogel-based bioinks).^11^ Cell-based bioinks comprise cell suspensions or aggregates, and cell spheroids, whereas hydrogel-based bioinks include cell-laden natural, synthetic, and decellularized tissue hydrogels.^12–15^ Bioinks usually lead to mechanically weak constructs that could hardly self-support themselves, which significantly hinders the complex bioprinting of human-scale constructs.^16–18^ In addition, native tissues are multicellular that comprise many cell types, which require the ability to formulate and bioprint multiple cell types while maintaining their phenotype or derive them into site specific lineages. Moreover, fabrication of vascular networks that are embedded within the bioprinted tissues is one of the key issues to achieve large-scale bioprinting as vasculature is crucial for nutrient supply and waste removal to overcome mass transfer limitations.^19,20^

Although a wide range of 3D printing technologies is currently accessible, many of these technologies are not suitable for bioprinting due to cell friendly processing requirements for live cell printing.^21–23^ Bioprinting technologies include extrusion-based direct ink writing (DIW), droplet-based inkjet printing,^24^ and laser-induced forward transfer (LIFT),^25^ and vat polymerization-based bioprinting including stereolithography (SLA), digital light processing (DLP), and continuous digital light processing (cDLP). Among these bioprinting techniques, DIW is the most commonly used technology due to ease of use, availability, and low cost. DIW's ability to fabricate multimaterial constructs is a significant advantage over vat polymerization printing methods. It can also work with a wider range of materials than vat-based systems. Using a digitally controlled pneumatic or mechanical print head, bioinks could be extruded through a nozzle to stack them layer-by-layer that undergo a certain cross-link process to gain structural integrity.^26,27^ In a droplet-based bioprinting process, microdroplets are created and projected onto the substrate to create constructs in a layer-by-layer manner.^28–30^ Droplet-based bioprinting is intrinsically easy to precisely deposit bioinks, but the printed droplets need a rapid liquid–solid transition to be structural integrated and self-supportive. Vat-polymerization-based bioprinting utilizes a light source (e.g., laser beam, laser projection, or two-photon laser) to selectively cross-link the cell-laden photocurable polymer solution.^31–33^ The print resolution (the minimum printable object size), the print speed (the required print time), and the number of compatible bioink materials are the key factors to consider when selecting the most suitable bioprinting technology for tissue and organ printing. For instance, the resolution of the extrusion-based bioprinting is mainly determined by the bioink and nozzle size, which limit the maximum resolution to ∼100 *μ*m.^34^ For the droplet-based bioprinting, print resolution is approximately 25–50 *μ*m,^21^ whereas for vat polymerization-based bioprinting, the resolution is in the range of 10–100 *μ*m.^31,33,35^ The advancement of multi-photon technology has enabled nano-scale resolution.^36^ The extrusion-based bioprinting has relatively higher speed when compared to droplet-based and most of the vat polymerization-based bioprinting techniques.^21,34^ However, increasing the printing speed in extrusion-based bioprinting usually leads to decrease in resolution. Each bioprinting technology has a different requirement for bioinks. For extrusion-based bioprinting, the bioinks should fulfill the physicochemical requirements, among which rheological properties (viscosity, viscoelastic shear moduli, elastic recovery, shear stress, etc.) are the most critical to determine the printability of the bioinks.^37^ The bioink viscosity is typically in the range of 30–6 × 10^7^ mPa·s, whereas for highly viscous materials, shear-thinning property is required to mitigate the high shear stress during the extrusion. The extruded ink also needs to be stabilized by engineered cross-link strategies, including pH or temperature changes, ionic cross-linking, photochemical reactions, enzymatic cross-linking, and guest−host interactions.^37^ Droplet-based bioprinting requires lower viscosity, which is in the range of 1–300 mPa·s.^16^ In particular, surface tension, density, and volatility are important parameters for droplet-based bioprinting.^38,39^ For vat polymerization-based bioprinting, the typical viscosity of the bioink is around 250–10^4^ mPa·s, while the optical (light transparency and absorbency) and photopolymerization properties are more critical than physiochemical properties.^40^ Most of the vat polymerization systems are based on chain growth and thiol-ene step-growth polymerization reactions, yet some of non-covalent cross-linking mechanisms are also available for vat polymerization.^37,41^

For complex 3D bioprinting, two aspects of complexities of the printed tissue/organ constructs are usually considered to resemble *in vivo* conditions, including the tissue architecture and the physical (stiffness) and biochemical (cells and bioactive cues) complexity. Due to layer-by-layer fabrication, 3D printing of complex architectures such as tubular and spiral as well as hollow structures (such as embedded channels for vascularization) is limited. To resemble the physical and biological complexity, it is crucial to place a multitude of bioinks within a 3D space, allowing precise distribution of multiple cell types and extracellular matrix (ECM) mimetic materials. Recent advances in bioprinting technology and bioink development enabled to overcome some of the above-mentioned issues, yet there is still more to accomplish to achieve fabrication of fully functional, human-scale, and highly complex tissues and organs. In this review, we summarize the evolution of complex 3D bioprinting from simple gel-casting to multi-material bioprinting to current state-of-the-art omnidirectional bioprinting approaches and emerging 4D and *in situ* bioprinting technologies. We discussed each technology in depth including the advantages and disadvantages toward complex 3D bioprinting of human-scale tissues and organs. We hope that this review will provide insight and foresight of the developments in this field and help researchers to find new ideas and opportunities.

## EVOLUTION OF COMPLEX 3D BIOPRINTING

### Inkjet bioprinting

**FIG. 1.**
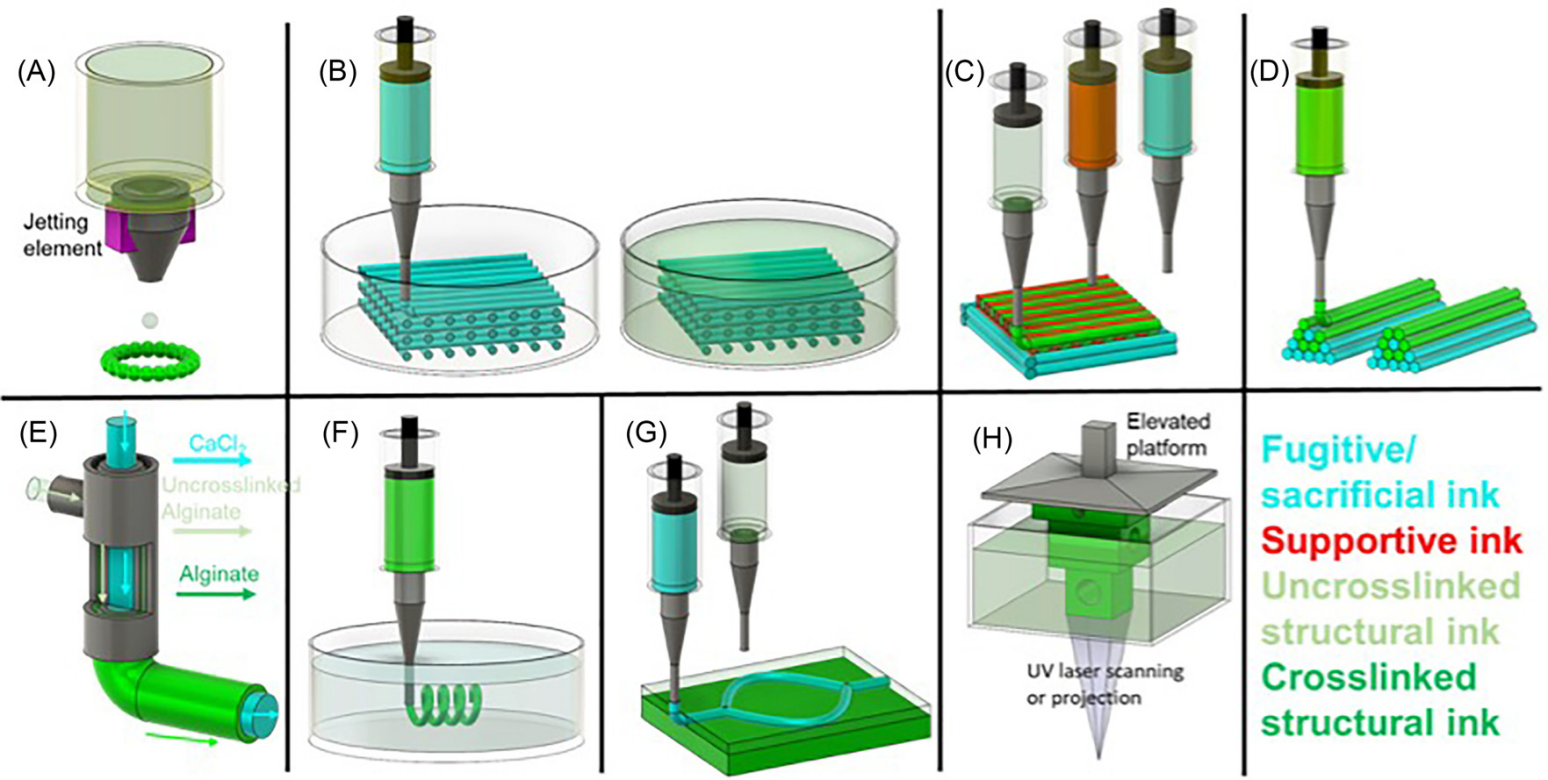
The evolving of complex bioprinting: (a) inkjet bioprinting; (b) 3D printing integrated with gel-casting; (c) 3D bioprinting with support materials; (d) unit-stacking approach; (e) coaxial bioprinting; (f) freeform bioprinting; (g) sequential freeform bioprinting; (h) light-assisted bioprinting introduced.^92^

Inkjet bioprinting uses a jetting element (i.e., a thermal or a piezoelectric actuator) to generate and dispense droplets on a substrate [Fig. 1(a)]. In 2003, the first bioprinting experiment was performed by Boland and co-workers, which started the whole bioprinting field.^42,43^ A commercially available 2D inkjet printer was modified to dispense protein or cell suspensions. To achieve 3D fabrication, a layer of poly[N-isopropylacrylamide-co-2-(N,N-dimethylamino)-ethyl acrylate] (PNIPAM)-based ink was dispensed on a collagen substrate, whereas cell suspensions [bovine aortal endothelial cells (bECs)] were jetted on the PNIPAM layer, and this sequential step was repeated, leading to an increase in printed construct thickness.^43^ The cell attachment, migration, and viability were evaluated, from which the results showed cells survived after jetting, and the cells were able to spread, migrate, and fuze together on the surface of a PNIPAM layer. These early trials demonstrated the feasibility of cell printing. The same group also simultaneously bioprinted human microvascular endothelial cells and fibrin to form microvasculature.^44^ These attempts were pioneering in the bioprinting field; however, the printed construct lacked structural and cellular complexity due to the non-self-supportive nature of the aqueous cell suspensions, which urged researchers to find approaches to enable inkjet printing of self-supportive cellular constructs. In this regard, Xu *et al.* bioprinted heterogeneous multicellular tissue constructs by separately dispensing calcic cell suspensions [human amniotic fluid-derived stem cells (hAFSCs), canine smooth muscle cells (dSMCs), and bovine aortic endothelial cells (bECs)] into a bath containing alginate/collagen, which allowed rapid gelation of the bioinks.^45^ For commercially available inkjet printers, the majority of the inks must render low-viscosity or specific electromagnetic properties, significantly limiting the usage of inkjet bioprinting. Recently, Foresti *et al.* developed a novel jetting system that utilized acoustophoretic force to drive the formation of the droplets, which enabled jetting a broad range of soft materials, including high viscosity Newtonian fluids (up to 25 000 mPa·s) and yield stress fluids (τ_0_ > 50 Pa).^46^ Specifically, the authors printed hMSC-laden collagen I bioinks on glass slides followed by a secondary hydrogel matrix encapsulation and culturing. During the culturing stage, the human mesenchymal stem cells (hMSCs) showed high viability (>80%) and proliferation within the droplet region, while the stemness was retained. In summary, inkjet bioprinting has the advantage in printing multiple bioinks, but direct inkjet bioprinting of self-supportive tissue constructs is still challenging, and the lack of commercially available inkjet bioprinters significantly limits the popularization of inkjet bioprinting.

### 3D printing integrated with gel-casting

**FIG. 2.**
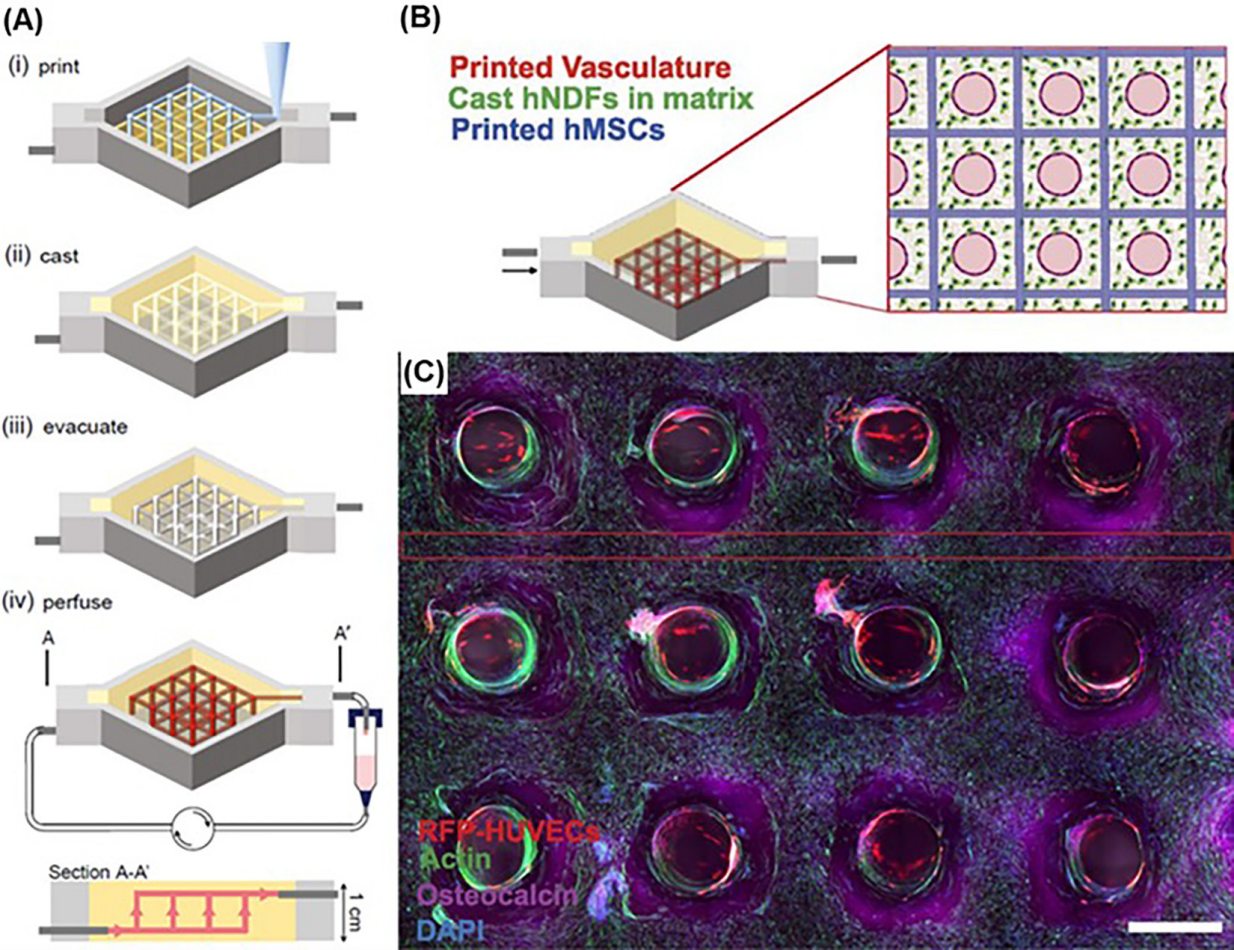
Constructing a thick tissue by casting hydrogels over 3D printed sacrificial structures:^50^ (a) Schematic illustration of the 3D printing and gel-casting process; (b) schematic description of the printed heterogeneous tissue within the perfusion chip, wherein the branched vascular architecture pervades hMSCs that are printed into a 3D lattice architecture, and HNDF-laden hydrogels were cast to fill the void space in the chip. (c) Confocal microscopy image through a cross section of the vascularized tissue construct after 30 d of active perfusion and *in situ* osteogenesis. (scale bar: 1.5 mm). Adapted with permission from Kolesky *et al.*, “Three-dimensional bioprinting of thick vascularized tissues,” Proc. Natl. Acad. Sci. U.S.A. **113**(12), 3179–3184 (2016). Copyright 2016 Authors, licensed under a Creative Commons Attribution (CC BY) License.

An early example of complex 3D bioprinting involves 3D printing of sacrificial structures integrated with the hydrogel casting process [Fig. 1(a)]. This approach includes mold fabrication, 3D printing of sacrificial structures within the mold, hydrogel precursor solution casting into the mold followed by hydrogel cross-linking, and finally removal of the sacrificial structure.^47–53^ This approach is one of the widely used strategies for fabrication of channel structures embedded within 3D cell-laden hydrogels. For example, Miller *et al.* printed a carbohydrate glass lattice as a sacrificial structure for gel-casting a series of cell-laden hydrogels, including agarose, alginate, PEG, fibrin, and Matrigel. The human umbilical vein endothelial cells (HUVEC) were seeded on the channel surface after the sacrificial structure is removed. These channels were then perfused with culture media. The cast hepatocyte-laden hydrogel sustained the metabolic function of the cells under perfusion.^47^ Kolesky and co-workers fabricated vascularized, heterogeneous tissue constructs by casting gelatin methacrylate (GelMA) into a mold housing a preprinted human neonatal dermal fibroblast (HNDF) or 10T1/2 MF-laden GelMA, and sacrificial Pluronic (Pluronic F127) structures. After the construct was exposed to UV light to cross-link GelMA, Pluronic was removed forming channels. These channels were then seeded with HUVECs for vascularization.^48^ Further advancing this approach, Kolesky *et al.* showed feasibility of printing a thick (over 1 cm in thickness) vascularized tissue that enables long-term perfusion (over 6 weeks) (Fig. 2).^50^ Human mesenchymal stem cell (hMSCs)-laden hydrogels were printed along with the sacrificial material, and HNDFs were embedded within the cast GelMA. HUVECs were then seeded after cross-linking of the GelMA and the removal of the Pluronic, forming a confluent endothelial cell layer. When the tissue was perfused with osteogenic induction media, osteogenic differentiation of hMSCs was achieved indicated by enhanced alkaline phosphatase (ALP) expression, Alizarin-Red staining, and osteocalcin staining.^50^ Apart from fabricating vascularized tissues, this approach was utilized to fabricate renal and bile tissues.^49,52,53^ To date, gel-casting is still widely used due to its cost-efficiency and ease to use.^54^ However, it is difficult to place channels at a user-defined height, unless there are multiple steps of sacrificial material printing and gel-casting.

### 3D bioprinting with support materials

**FIG. 3.**
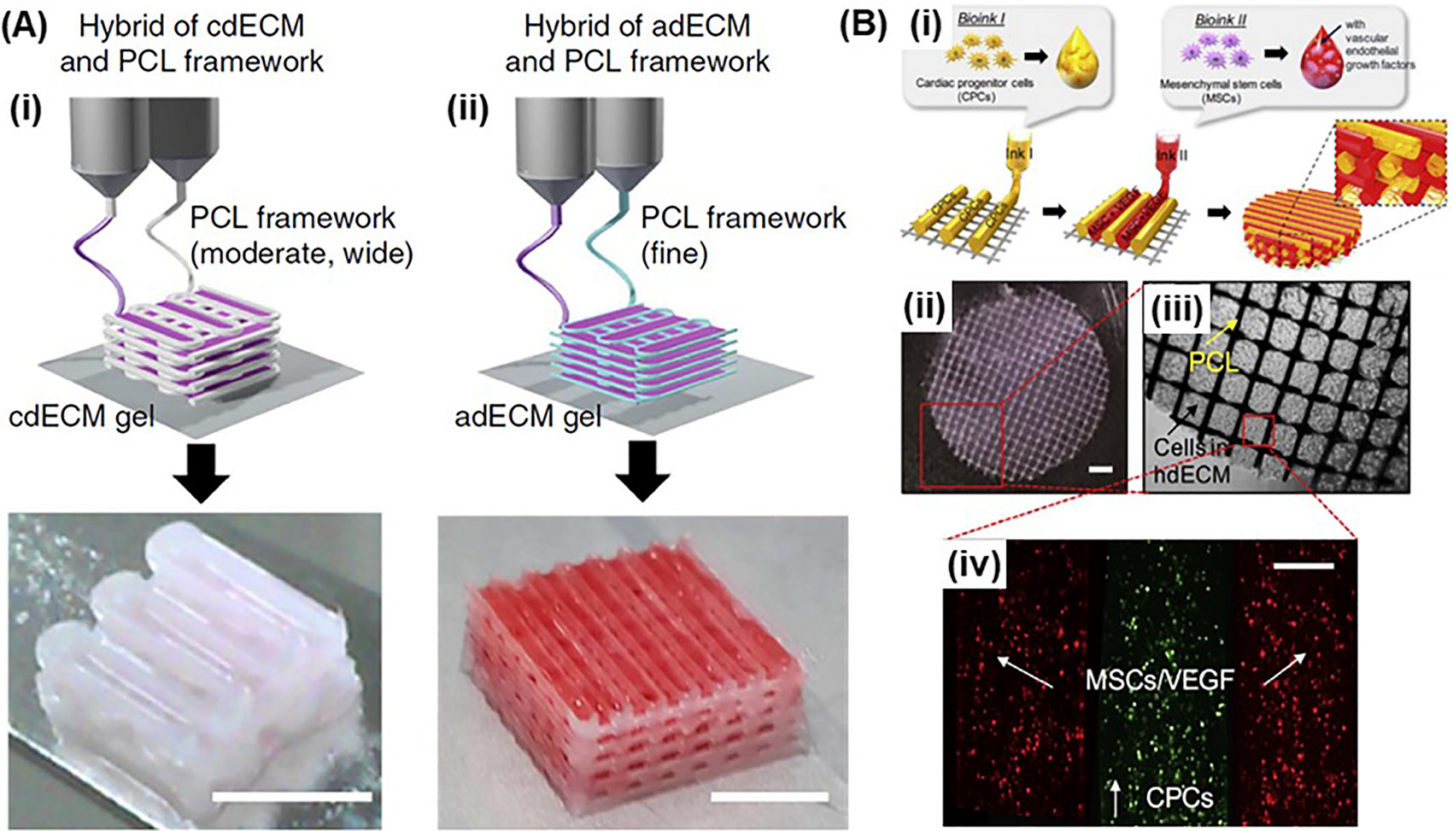
3D bioprinting with support materials: (a) 3D bioprinting structures with corresponsive dECM-based bioinks using PCL support: (i) cartilage tissue and (ii) adipose tissue. (scale bar: 5 mm) Adapted with permission from Pati *et al.*, “Printing three-dimensional tissue analogues with decellularized extracellular matrix bioink,” Nat. Commun. **5**, 3935 (2014). Copyright 2014 Authors, licensed under a Creative Commons Attribution (CC BY) License. (b) 3D bioprinting of pre-vascularized stem cell patch with multiple cell-laden bioinks supported by PCL. (scale bars: 1 mm for left mid; 200 *μ*m for bottom). Reprinted with permission from Jang *et al.*, “3D printed complex tissue construct using stem cell-laden decellularized extracellular matrix bioinks for cardiac repair,” Biomaterials **112**, 264–274 (2016). Copyright 2017 Elsevier.

One of the major obstacles that hinder 3D bioprinting of human-scale tissues and organs is our ability to fabricate self-supporting structures using bioprinting. Although the vat can provide support during printing in vat photopolymerization-based printing, support structures are required for extrusion-based DIW and droplet-based bioprinting processes to achieve large-scale constructs and constructs containing hollow structures.^55–59^ This requires printing of a support ink along with the bioink [Fig. 1(b)]. Support ink can be a sacrificial hydrogel such as Pluronic, which can be removed after printing, or a thermoplastic [such as polycaprolactone (PCL) and polyurethane (PU)] that can remain to provide mechanical stability for the bioprinted structure both *in vitro* and *in vivo*. In this regard, the Atala group developed an integrated tissue organ printer (ITOP) to fabricate complex tissue interfaces as well as vascularized cellular constructs that are clinically relevant in size, shape, and structural integrity.^55,56^ The process utilized an extrusion-based DIW with multiple print heads integrated with melt printing. Bioprinting cell-laden hydrogels and sacrificial hydrogels along with biodegradable polymers in integrated patterns enabled mechanical stability. Diffusion of nutrients was ensured by incorporation of microchannels. This approach was used to demonstrate bioprinting of complex tissue interfaces such as muscle-tendon interface comprising a muscle and a tendon phase that are integrated together.^55^ The muscle phase was bioprinted using C2C12 myoblasts-laden hyaluronic acid (HA)/gelatin/fibrinogen composite hydrogel along with polyurethane (PU) support material. NIH/3T3 fibroblast-laden composite hydrogel was bioprinted along with polycaprolactone (PCL) to fabricate the tendon phase. After the printing process, the construct was immersed in a thrombin/CaCl_2_ solution to cross-link the hydrogel. This approach enabled fabrication of the muscle-tendon constructs with spatially distinct mechanical properties, such that the Young's modulus of the tendon phase (E = 46.67 ± 2.67 MPa) was ∼120-folds higher than the muscle phase (E = 0.39 ± 0.05 MPa).^55^ Authors also demonstrated fabrication of mandible and calvarial bone, cartilage, and skeletal muscle.^56^ Cell-laden hydrogel is bioprinted along with PCL (or PCL composite) support material, and sacrificial pluronic hydrogel (pluronic F127) was printed as a contour to confine the shape of the cell-laden hydrogel. The bioprinted thick tissue constructs with more interconnective pores showed enhanced cell viability and vascularization. When implanted, bioprinted tissue constructs showed robust tissue formation, such as bone, muscle, and cartilage.^56^ A similar strategy was used by Pati *et al.* to bioprint decellularized extracellular matrix (dECM)-based bioinks to fabricate adipose (fat), cartilage, and heart tissue analogs [Fig. 3(a)].^60^ Jang *et al.* bioprinted cardiac patches composed of dECM hydrogels supported with PCL [Fig. 3(b)].^57^ The dECM hydrogels encapsulated human c-kit^+^ cardiac progenitor cells (hCPCs), or hMSCs, or combination of the two cell lines that were supplemented with a vascularization growth factor and vitamin B_2_ as a crosslinker. The bioprinted patches showed therapeutic efficacy through enhancement of cardiac function and decrease in negative regulation of tissue remodeling *in vivo*.^57^

While bioprinting with support materials enable construction of complex-shaped tissues, commonly used thermoplastic support materials, such as PCL, require elevated temperatures (60 °C or higher) for printing, and they have significantly stronger mechanical properties as compared to cell-laden hydrogels, which may not be suitable for many soft tissues. Besides, the thermoplastics need months or even years (1.5–2 years for PCL) to degrade *in vivo*,^61^ which provides enhanced structural stability but could impede the rapid regeneration and growth of the tissue. The achievable size and complexity are also common issues when sacrificial hydrogels are printed as support.

### Unit-stacking approach

**FIG. 4.**
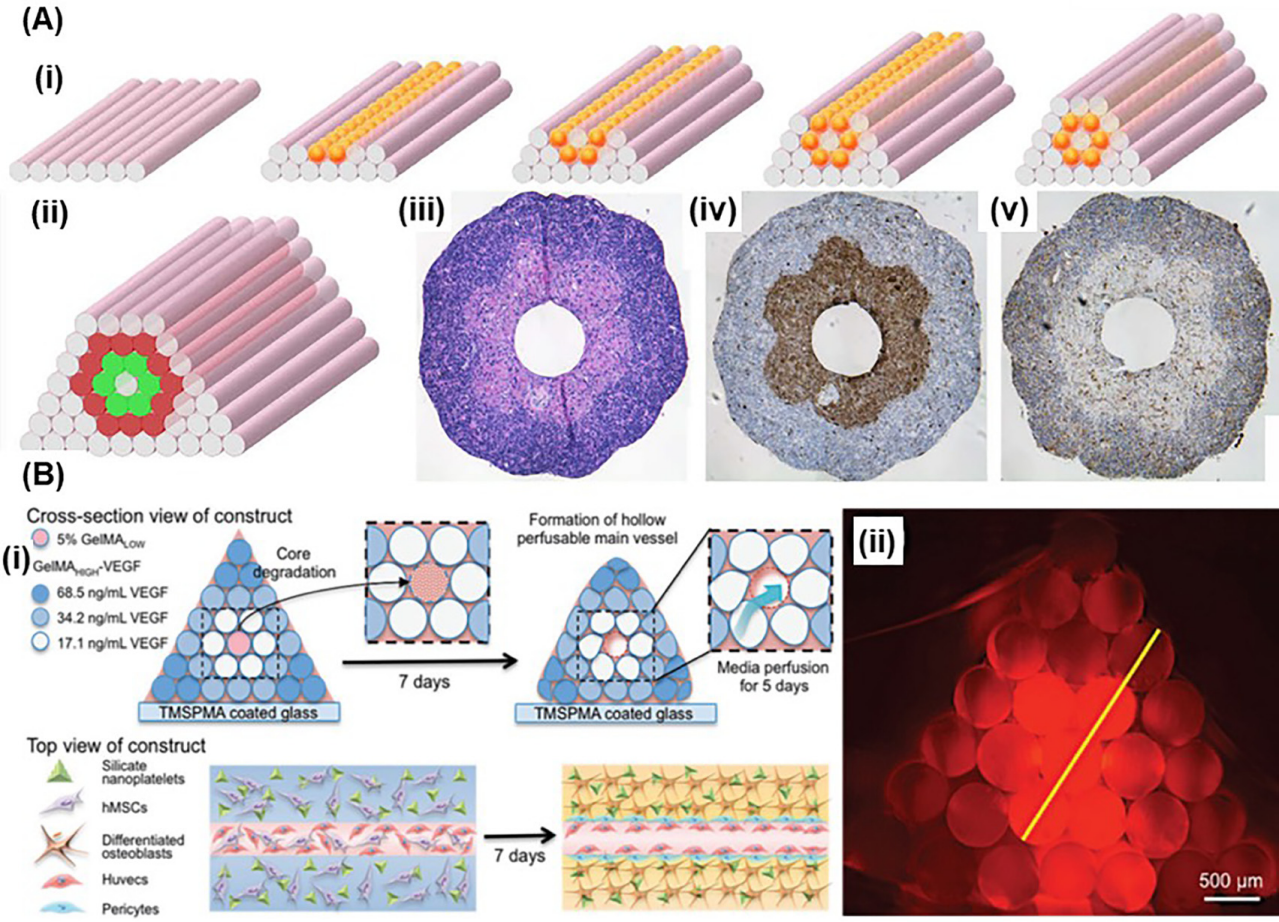
Unit-stacking approach: (a) (i) Constructing tubular structure by stacking sacrificial (pink) and cellular (orange) units; (ii) building a double-layered vascular wall by stacking sacrificial (pink) and cellular (green for smooth muscle cells, red for skin fibroblasts). (iii)–(v) show the histological examination results after 3 days of fusion: H&E (iii), smooth muscle a-actin (brown; iv), and Caspase-3 [brown; (v)]. Reprinted with permission from Norotte *et al.*, “Scaffold-free vascular tissue engineering using bioprinting,” Biomaterials **30**, 5910–5917 (2009). Copyright 2009 Elsevier. (b) (i) Constructing bone mimetic 3D tissue containing osteogenic and vasculogenic units; (ii) cross-section image of the pyramidal stacked cylinders (stained with Texas Red). Reprinted with permission from Byambaa *et al.*, “Bioprinted osteogenic and vasculogenic patterns for engineering 3D bone tissue,” Adv. Healthcare Mater. 6, ▪ (2017). Copyright 2017. John Wiley and Sons.

In unit-stacking approach, cell-laden hydrogels are bioprinted as cylinders or spheroids, which serve as the building units that can be stacked into a desired shape or a construct [Fig. 1(c)].^62–67^ For instance, Jakab *et al.* extruded cell aggregates through a capillary tube forming sausage-like cellular cylinders, which were cut into fragments that were rounded into spheroids.^62^ These spheroids were then bioprinted into 3D constructs up to 12 layers that were completely fused together in 6 days. The printed cardiac tissue (from chicken cardiomyocytes) was functional and able to synchronously beat after 90-h of culture.^62^ Unit stacking is effective to bioprint tubular structures, which is difficult for extrusion-based bioprinting to achieve when using a single material. For instance, Norotte and co-workers prepared cell-laden cylinders and spheroids as building units to bioprint vessel-like tissue constructs (with a diameter of 0.9 mm to 2.5 mm) to mimic blood vessels [Fig. 4(a)].^64^ Cell-laden units and supportive (agarose) units were deposited by extrusion-based bioprinting and stacked to form linear and branched vessels. Linear vessels were bioprinted using cylinder, whereas spheroids were used for branched vessels. The stacked constructs were cultured for up to 7 days to allow for the self-assembly of the cell-laden units, and the agarose support was removed.^64^ Skardal *et al.* fabricated sausage-like hydrogel macrofilaments with high cell density (NIH 3T3, up to 25 M/ml) using thiolated hyaluronic acid (HA) and gelatin that are co-crosslinked with four-armed PEG tetracrylates.^68^ Agarose macrofilaments were used as a support. These macrofilaments were bioprinted (syringe based Fab@Home printing system). Agarose support was removed after the stacked macrofilaments fused during 28 days culture, forming a vessel-like structure.^68^ Unit-stacking approach was also used to fabricate vascular bone tissue using cylindrical units of GelMA-based hydrogels [Fig. 4(b)].^67^ Unit-stacking approach is efficient for bioprinting straight tubular structures in macroscale. The resolution of the bioprinting is determined by the dimensions of the stacked units. Building units with finer size could help to bioprint tissue constructs with much higher complexity.

### Coaxial bioprinting

**FIG. 5.**
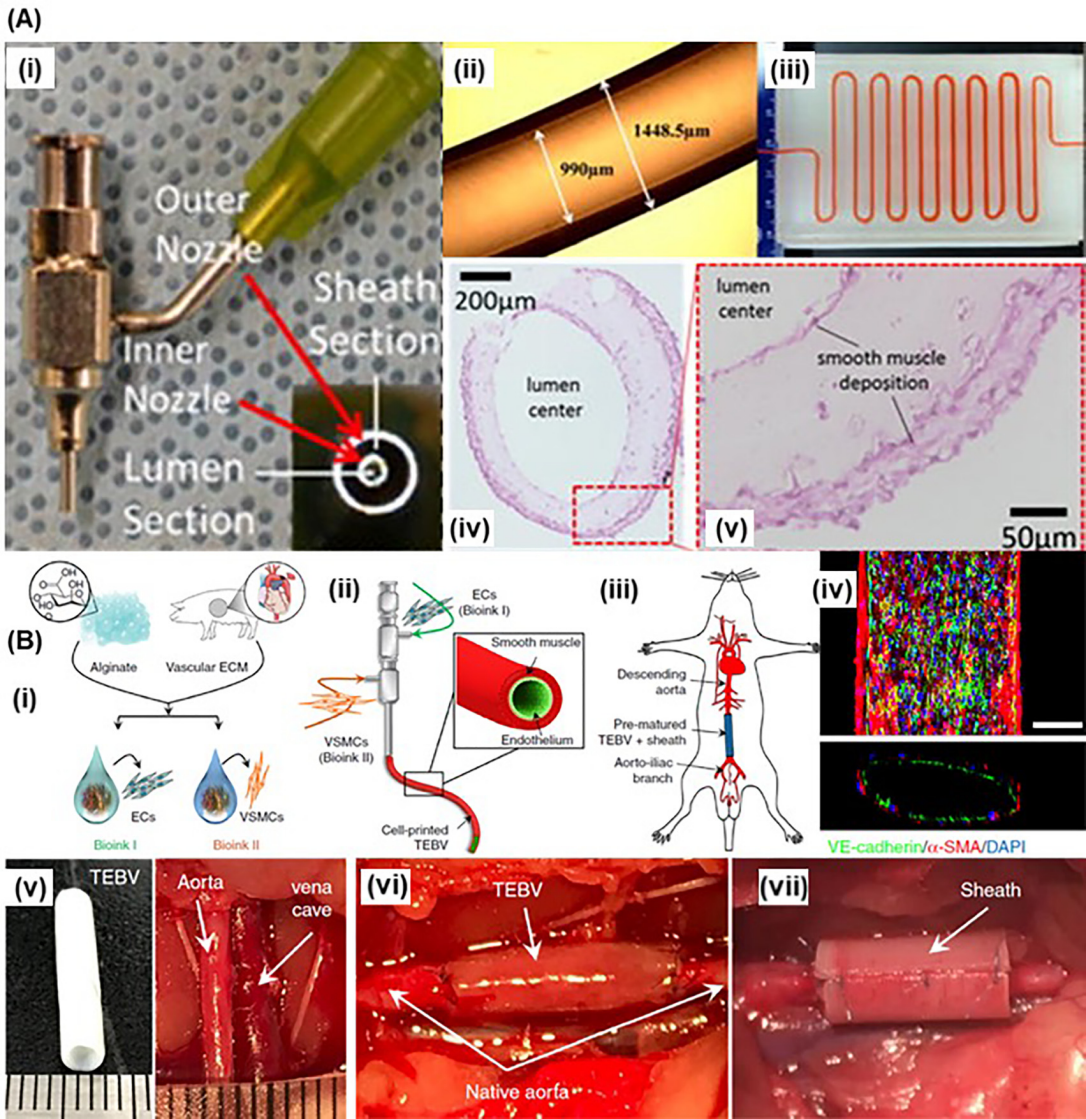
Coaxial bioprinting: (a) (i) a homemade coaxial nozzle unit; (ii) light microscope image of conduits; (iii) perfusing a long vasculature conduit with cell culture media; (iv) and (v) a light microscope image of a cross section slice that Verhoeff–Van Gieson staining (light pink color) showed smooth muscle matrix deposition around cells and throughout the conduit wall. Republished with permission from Zhang *et al.*, “In vitro study of directly bioprinted perfusable vasculature conduits,” Biomater. Sci. 3, 143–145 (2015). Copyright 2015 Royal Society of Chemistry (b) 3D bioprint TEBV containing endothelium and smooth-muscle using triple-coaxial cell printing: (i) bioink formulation; (ii) schematic illustration of the triple-coaxial printing; (iii) *in vivo* evaluation of prematured TEBVs through implanting abdominal aorta graft in a rat model; (iv) confocal microscopic image of bioprinted blood vessel, which combined the endothelial (green) and muscular (red) layers (scale bar: 200 *μ*m); (v) the prematured TEBV had a similar dimension to rat abdominal aorta; (vi) implanting TEBV as (vi) interposition grafts with (vii) an added polycaprolactone (PCL) sheath. Reprinted with permission from Gao *et al.*, “Tissue-engineering of vascular grafts containing endothelium and smooth-muscle using triple-coaxial cell printing,” Appl. Phys. Rev. **6**, 041402 (2019). Copyright 2019 AIP Publishing.

Coaxial bioprinting is suitable for continuous fabrication of tubular structures. The key feature of coaxial bioprinting is the two-layered nozzle, which enables co-extrusion of two different bioink formulations in a core-shell manner [Fig. 1(d)].^69–71^ Tubular structures are not geometrically complex, yet they are very difficult to fabricate by 3D bioprinting. In 2013, Ozbolat group used Luer-lock dispensing needles to fabricate coaxial nozzles for bioprinting, and demonstrated bioprinting of cartilage progenitor cell (CPC)-laden alginate tubing.^70^ For this purpose, CaCl_2_ solution was extruded in the core section which crosslinked the cell-laden alginate extruded in the shell layer. By controlling the bioink formulation, the dispensing pressure of alginate bioink, and the flow rate of the CaCl_2_ solution, it was possible to achieve cell-laden alginate hollow tubes with tunable inner and outer diameter (and wall thickness). The CPCs retained high viability, and the tubing retained the structural integrity after perfusion. In a following study, Yu *et al.* evaluated the cell viability and functionality in the bioprinted cell-laden tubular channels and demonstrated that lower dispensing pressure (35 kPa), larger coaxial nozzle size (730 *μ*m), and lower alginate concentration are in favor of higher cell viability.^72^ To reinforce the weak mechanical properties of alginate, multiwall carbon nanotubes (MWCNT) were used to reinforce smooth muscle cell-laden alginate bioinks to print vascular conduits, which increased the tensile strength by 11%, burst pressure by 6.5%, and elastic modulus by 94% without affecting short-term (1 week) cell viability.^73,74^ The MWCNTs were cytotoxic in long-term (6 weeks), while in plain alginate vascular conduits, most of the cells were intact and able to migrate, proliferate, and deposit smooth muscle matrix around the peripheral and luminal surface in long-term. Such ECM deposition was observed in another *in vitro* study, in which the dehydration, swelling, degradation, permeability, and mechanical properties of the bioprinted vascular conduits were investigated in detail [Fig. 5(a)].^75^ To fabricate scale-up tissues, Yu *et al.* developed a hybrid bioprinting approach to print fibroblast cell aggregate strands along with coaxial printed vasculatures. The cell strands fused in 7 days and attached to the vasculatures, which was perfusable for long-term culture.^76^

Due to the ease of direct bioprinting of tubular channels, researchers formulated endothelial cell-laden bioactive bioinks to fabricate thick, vascularized, functional tissues.^77–82^ For instance, Zhang *et al.* formulated a HUVEC-laden GelMA/alginate bioink to coaxially bioprint endothelialized organoids.^83^ The organoids were cultured in a medium to allow HUVECs to migrate and form confluent endothelium on the surface of the channels, and then seeded with cardiomyocytes and cultured in a perfusion bioreactor to finally make endothelialized-myocardium-on-a-chip platforms for cardiovascular toxicity evaluation. In another study, Cui *et al.* bioprinted self-standing, small-diameter (∼1 mm) vasculature with stratified architecture by coaxial extrusion.^79^ A smooth muscle cell-laden catechol-functionalized gelatin methacrylate (GelMA/C) bioink was extruded in the shell layer, while the fugitive cross-linking slurry containing pluronic F127, sodium periodate (NaIO_4_), and HUVECs was extruded in the core section to cross-link GelMA/C via rapid oxidative cross-linking *in situ*. After removing pluronic and endothelium formation, the vascularized tissue construct was immersed in an MSC-laden GelMA bath for SLA printing to fabricate a multicellular vascularized tissue model. Noticeably, *in vivo* evaluations of the bioprinted vascularized tissue constructs were performed in an immunodeficient murine model, which showed autonomous connection (∼2 weeks) and vascular remodeling (∼6 weeks). Gao *et al.* extracted vascular-tissue dECM (VdECM) from porcine aortic tissue and formulated a hybrid bioink platform using VdECM and alginate [Fig. 5(b)].^82^ The authors also developed a triple-coaxial bioprinting system that enabled direct extrusion of F127/CaCl_2_, HUVEC-laden VdECM/alginate, and human aortic smooth muscle cell (HAoSMC)-laden VdECM/alginate (from core section to outer layer) to fabricate tissue-engineered blood vessels (TEBVs) containing an inner layer of endothelial cells and an outer layer of muscular cells. After *in vitro* static and dynamic cultivation, the TEBVs were reported to achieve sufficient strength, cellular alignment, and contractile property. These TEBVs were further implanted as a graft in a rat abdominal aorta for 3 weeks, in which the grafts retained the structure and integrated with the host tissue.

**FIG. 6.**
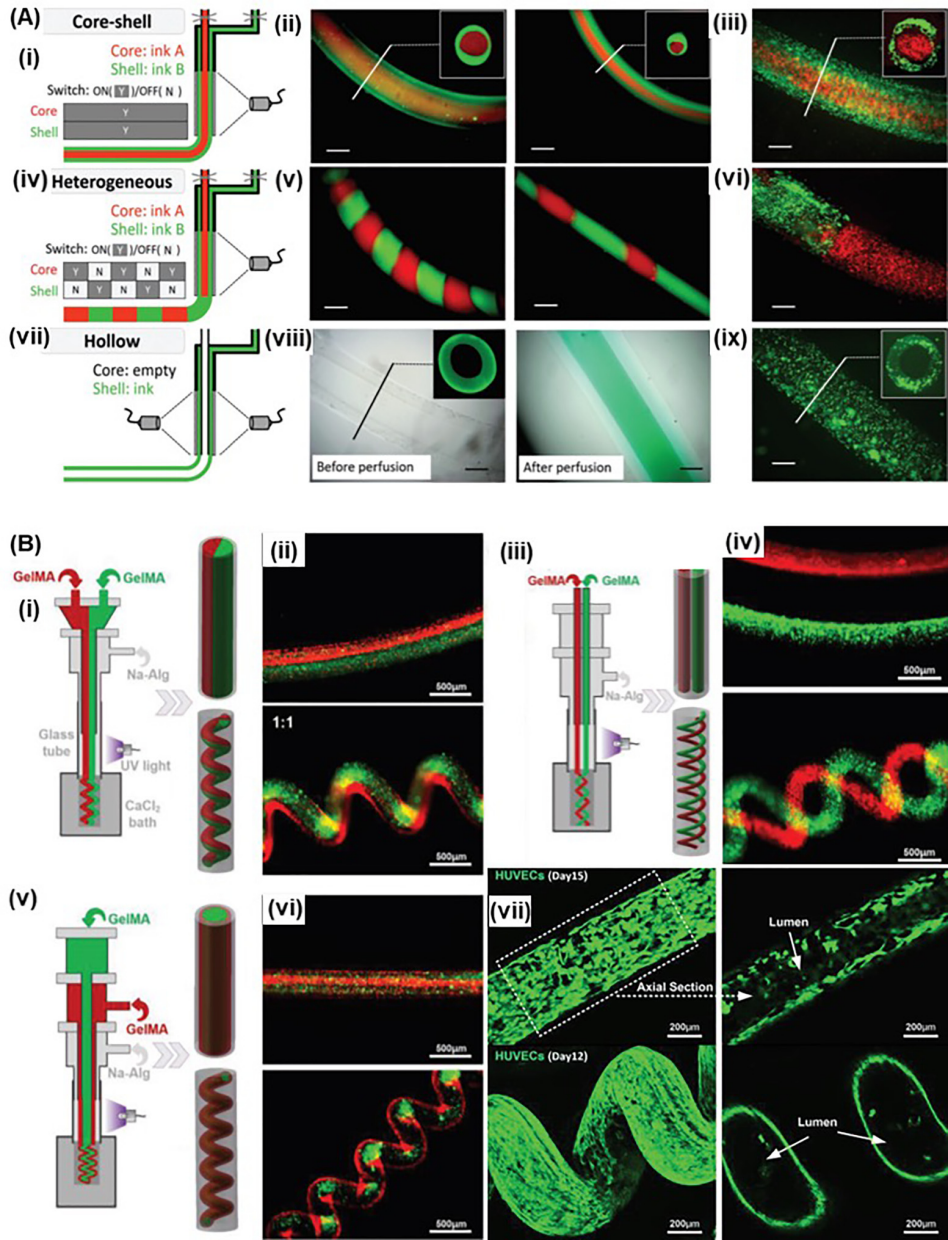
Novel coaxial bioprinting integrated with *in situ* cross-linking: (a) schematic and representative fluorescence images for printing filaments of different architectures: (i) core-shell structure printed with different fluorophores (ii) or two inks containing cells labeled with different dyes (iii); (iv) heterogeneous filaments with intermittent structures Schematic and representative fluorescence images for printing filaments printed with different fluorophores (v) or two inks containing cells labeled with different dyes (vi); (vii) hollow filaments printed by a longer core coaxial nozzle and representative images of printed hollow tubes either before or after perfusion with a dye solution (viii) or with cells in the printed tubes (ix). (scale bars: 500 *μ*m). Adapted from Ref. 84. Reprinted with permission from Ouyang *et al.*, “A generalizable strategy for the 3D bioprinting of hydrogels from nonviscous photo‐crosslinkable ink,” Adv. Mater. **29**, ▪ (2017). Copyright 2016 John Wiley and Sons. (b) Bioprinting the multicompartmental GelMA microfibers: (i) Schematic illustration of the printing of straight and helical GelMA microfibers with Janus structure; (ii) fluorescence microscopy images of GelMA microfibers with Janus structures; (iii) schematic illustration of the generation of double paratactic straight and double helical GelMA microfibers; (iv) fluorescence microscopy image of double paratactic straight and double helical GelMA microfibers; (v) schematic illustration of the printing of multilayered straight and helical GelMA microfibers; (vi) fluorescence microscopy image of double-layered straight/helical GelMA microfibers; (vii) the axial section of confocal laser-scanning microscopy images of the straight and helical GelMA microfibers showing the straight HUVECs lumen after 15 days of culture and helical HUVECs lumen after 12 days of culture. Reprinted with permission from He *et al.*, “Fiber‐based mini tissue with morphology‐controllable GelMA microfibers,” Small **14**, e1802187 (2018). Copyright 2018 John Wiley and Sons.

Recent studies showed coaxial bioprinting of heterogeneous and hollow filaments enabling fabrication of complex tissue constructs.^84–87^ For instance, Burdick group used a UV-transparent capillary tube that is connected to the end of the coaxial nozzle for *in situ* photocrosslinking, enabling bioprinting of nonviscous photocrosslinkable hydrogel-based bioinks [Fig. 6(a)].^84^ By controlling the flow of the inner and outer layer, core-shell, intermittent, and hollow tube structures were successfully bioprinted allowing fabrication of complex tissue constructs. Inspired by “liquid rope-coil effect,”^88,89^ Shao *et al.* used a similar coaxial/*in situ* photocrosslinking system to fabricate complex GelMA microfibers within a CaCl_2_ solution bath, where straight, wavy, and helical morphologies were achieved by controlling the flow rates [Fig. 6(b)].^87^ Moreover, Janus, multilayered, and double helix structures could be fabricated by changing the coaxial nozzle design. HUVECs were encapsulated in the shell and migrated toward peripheral of the microfibers and eventually formed endothelium, which resembled blood vessels.

Overall, the coaxial bioprinting is suitable for direct fabrication of tubular conduits with material complexity. However, limited by the nozzle size, the minimum achievable inner diameter of the printed tubing is around 500 *μ*m. In addition, these tubular conduits are difficult to integrate into 3D printed thick tissues and fabricate branching interconnected networks, which are limiting the widespread adoption of this approach.

### Freeform bioprinting

The above-mentioned approaches significantly enhanced bioprinted construct complexity and expand the bioprintable material formulations. However, the layer-by-layer printing process significantly limits the achievable complexity of the microstructures and 3D anisotropy as well as the ability to print tissue mimetic soft hydrogels (elastic modulus below 100 kPa) or cells alone. As described above, to print overhanging structures, soft hydrogels can be printed along with a support material to prevent collapse or deformation of the printed structure under its own weight, while printing such structures solely with soft hydrogels is still not possible. Freeform extrusion-based bioprinting overcomes these issues by eliminating the need for layer-by-layer fabrication and enabling omnidirectional freeform fabrication. In this approach, DIW is performed within a support bath which physically supports the printed structure [Fig. 1(e)]. The ink is extruded out of a needlelike nozzle that moves through a support bath, deposited within the bath, and held in place. Because the ink is embedded within the support bath, freeform bioprinting is also referred to as “embedded” bioprinting. Thus, support material needs to behave solid-like to provide physical support, yet it becomes fluid-like under applied shear stress to allow nozzle to move freely and recovers immediately when the stress is removed to hold the printed structure in place.^90^

Freeform fabrication is suitable for fabrication of 3D microvascular structures—interconnected microchannels,^91–93^ or 3D complex biological structures.^94–101^ To create 3D microvascular structures, a fugitive ink should be extruded within a support bath composed of a biologically relevant cell-laden hydrogel, organoid slurry, or a hydrogel precursor that can be crosslinked post-printing before the fugitive structure is removed. In a recent study, Skylar-Scott *et al.* developed sacrificial writing into a functional tissue (SWIFT) method to bioprint sacrificial gelatin inks within iPSC-derived organoid slurry, which enabled rapid fabrication of perfusable patient- and organ-specific tissues at therapeutic scales.^93^ On the other hand, 3D complex biological structures can be bioprinted using a cell-laden hydrogel (or hydrogel precursor) or a cell suspension as the ink and a fugitive material as the support. The removal of support can be triggered by thermally melting the support medium,^95,97,101^ altering the ion concentration/pH of the medium,^102^ enzymatic digestion,^99,100^ or by gently agitation.^98^

**FIG. 7.**
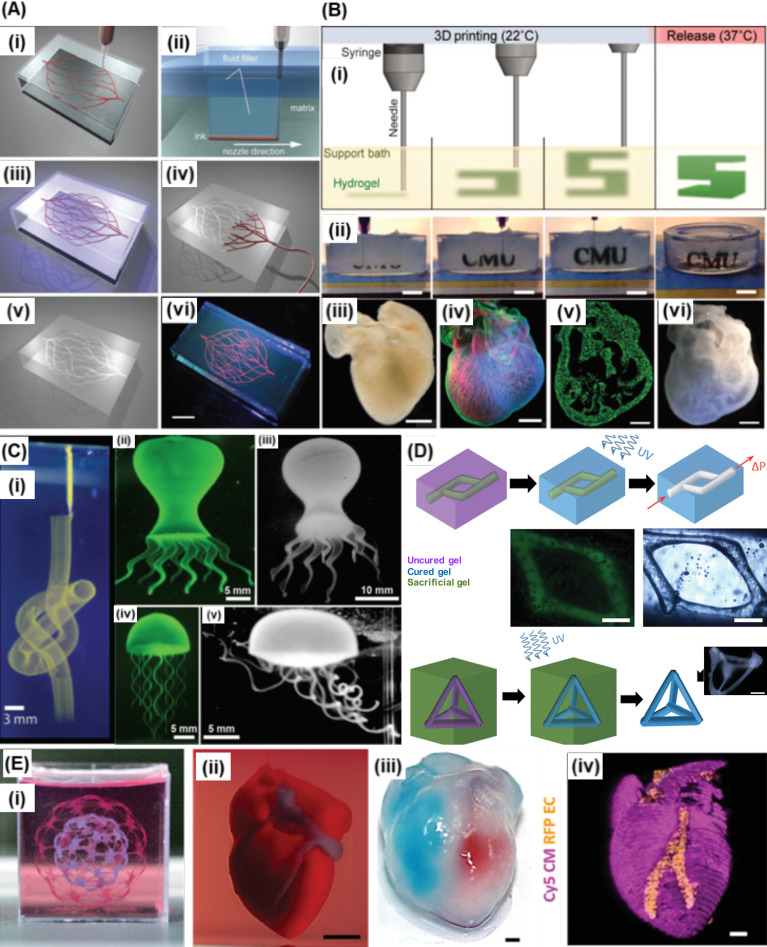
Freeform bioprinting approaches: (a) omnidirectional printing of 3D microvascular networks: (i) printing F127 network within the support bath; (ii) the fluidic layer filled the void space following the movements of the needle; (iii) cross-link the support bath with UV radiation; (iv) removing the F127 network; (v) omnidirectional hollow channels; (vi) perfusing the omnidirectional channels. (scale bar: 10 mm). Reprinted with permission from Wu *et al.*, “Omnidirectional printing of 3D microvascular networks,” Adv. Mater. **23**, H178–H183 (2011). Copyright 2011 John Wiley and Sons. (b) FRESH printing technique: (i) schematic illustration of the FRESH printing process; (ii) images of the letters FRESH printed in alginate and released by melting the gelatin support (gray material in the petri dish); (iii) a darkfield image of an explanted embryonic chick heart; (iv) a confocal microscope image of the chick heart stained for fibronectin (green), nuclei (blue), and F-actin (red); (v) a cross section image showing recreation of the internal trabecular structure of the 3D printed heart; (vi) a dark-field image of the 3D printed heart with internal structure visible through the translucent heart wall. [scale bar: 10 mm (ii), 1 mm (iii)–(vi)]. Adapted with permission from Hinton *et al.*, “Three-dimensional printing of complex biological structures by freeform reversible embedding of suspended hydrogels,” Sci. Adv. **1**(9), ▪ (2015). Copyright 2015 Authors, licensed under a Creative Commons Attribution (CC BY) License. (c) Writing in the granular gel medium: (i) a continuous tubing structure with multiple knots written within the support bath; (ii) a fluorescence image of a printed octopus after polymerization within the; (iii) an image of the octopus after removal from the support bath; (iv) and (v) a printed flexible jellyfish before and after removal from the support bath. Adapted with permission from Bhattacharjee *et al.*, “Writing in the granular gel medium,” Sci. Adv. **1**(8), e1500655 (2015). Copyright 2015 Authors, licensed under a Creative Commons Attribution (CC BY) License. Adapted from Ref. 94. (d) Printing channel structures or 3D frameworks by direct printing shear-thinning hydrogels into self-healing hydrogels. (scale bars: 500 *μ*m). Reprinted with permission from Highley *et al.*, “Direct 3D printing of shear‐thinning hydrogels into self‐healing hydrogelserials,” Adv. Mater. **27**, 5075–5079 (2015). Copyright 2015 John Wiley and Sons. (e) 3D printing personalized hydrogel in a supporting medium: (i) “Spheres in sphere” structure; (ii) a printed heart within a support bath; (iii) visualizing the left and right ventricles of the heart by injecting red and blue dyes; (iv) a confocal image of the printed heart (cardiomyocytes in pink, endothelial cells in orange). [scale bars: 0.5 cm for (ii), 1 mm for (ii)–(iv)]. Adapted with permission from Noor *et al.*, “3D printing of personalized thick and perfusable cardiac patches and hearts,” Adv. Sci. **6**(11), 1900344 (2019). Copyright 2019 Authors, licensed under a Creative Commons Attribution (CC BY) License.

In this regard, Lewis group developed an omnidirectional printing technique to fabricate 3D biomimetic microvascular networks [Fig. 7(a)].^91^ Photocurable Pluronic F127 diacrylate (F127-DA) was used as either a physical gel support material or a liquid capping layer on top of the support material, of which the rheological property was tailored by the F127-DA concentration. During the printing process, the nozzle translated through the uncured pluronic F127-DA support gel to deposit fugitive ink as desired vascular patterns, while the void spaces induced by the nozzle translation were filled with the liquid from the capping layer. After printing the fugitive ink, the whole construct was exposed to UV for cross-linking, and the fugitive ink was dissolved and removed by vacuum to form the microvascular channels.

Freeform bioprinting has been frequently used to fabricate 3D complex biological structures in the past five years. Feinberg group developed freeform reversible embedding of suspended hydrogels (FRESH) technique to direct-write hydrogels (elastic modulus < 500 kPa) within a thermoreversible support bath composed of gelatin microparticle slurry that supported the printed structure during printing and was melted at 37 °C post-printing [Fig. 7(b)].^95^ The FRESH technique is capable of printing collagen, alginate, Matrigel, and fibrinogen with or without cells, into complex shapes, including femurs, branched coronary arteries, trabeculated embryonic hearts, and human brains.^95^ Their versatile technique also allowed fabrication of devices from silicone (Sylgard 184 PDMS) within a Carbopol support bath, such as helix, tube, helical tube, perfusable tube, and perfusable bifurcations.^102^ In a following study, the same group focused on bioprinting collagen to engineer human heart components that replicated patient-specific anatomical structures with mechanical integrity and biological functionality.^97^ By optimizing the preparation of the support bath, the authors increased the resolution to reliably print collagen struts of ∼20 *μ*m in diameter. In this study, artery–scale linear tubes, porous collagen disks, cardiac ventricles, tri-leaflet heart valves, and adult human hearts were printed at remarkable accuracy. Noticeably, the printed small-scaled (4 mm) cardiac model ventricles were contractile, which were printed with collagen as structural material and high density human cardiomyocytes/human ventricular cardiac fibroblasts-laden fibrinogen as an infill. After 4–7 days of culture, the printed ventricles spontaneously beat at 0.5 Hz, or beat at 1 or 2 Hz under field stimulation. Note that the bioprinted ventricle model is small-scale, and the construction of a human scale cellularized, contractile, and viable heart remains unsolved. Bhattacharjee *et al.* used granular carbopol microgel as a support material that rendered rapid fluid to solid transition [Fig. 7(c)].^94^ They demonstrated printing of a wide range of materials, including silicone, colloids, hydrogels, and cells, to fabricate complex spiral, spheroidal, and shelled structures. The same group also demonstrated bioprinting of cells (dispersed in hyaluronidase) with 11 different cell types into arbitrarily complex structures within the liquid-like solid (LLS) material, in which the LLS material worked as both support and growth medium.^96^ Cell spheroids retained high viability (∼94%) after 24 h post-printing and retained defined shape with physical integrity in 2 weeks. Printed cell spheroids could be printed as a 3D array, which enabled high-throughput combinatorial multiple bioactive agents (i.e., combinatorial effects of two drugs) screening.

In a recent study, Dvir group bioprinted cardiac patches and hearts using patients' own cells and tissues [Fig. 7(e)].^99^ Cells were collected from a biopsy of an omental tissue taken from patients, reprogramed to become iPSCs, and differentiated to cardiomyocytes and endothelial cells, whereas the extracellular matrix of the biopsy was extracted and formulated with the obtained cardiomyocytes and endothelial cells as the two different personalized bioinks. To bioprint the personalized bioinks, the authors developed a fully transparent, cell-friendly, enzymatically/chemically degradable microparticulate support medium composed of alginate and xanthan gum. Blood vessels within thick tissues and small-scale cellularized human hearts mimicking the native architecture and stiffness were bioprinted. This study demonstrates the strong potential of the freeform approach to engineer personalized tissues and organs. Burdick group developed supramolecular hydrogels to suspend printed materials using reversible intermolecular bonds.^103,104^ Hyaluronic acid (HA) was conjugated with either adamantane (Ad) or β-cyclodextrin (CD), providing guest–host interactions between Ad and CD groups that rendered superb shear-thinning and self-healing properties,^105^ which allowed the extrusion of the inks into Ad-HA/CD-HA support gels to directly write structures continuously in 3D space [Fig. 7(d)].^103^ These intermolecular bonds allow for precise tailoring of the ink or the matrix rheology and can convert a viscous ink into a viscoelastic yield stress ink, which is ideal for embedded printing. This chemical modification strategy could be applied to many different polymers and macromers. Ad-HA and CD-HA could also modify with methacrylic groups to yield Ad-MeHA/CD-MeHA, which introduced photocrosslinking for irreversible cross-linking and better mechanical stability. By switching Ad-HA/CD-HA Ad-MeHA/CD-MeHA to be used as either structural ink or supportive bath, microchannels or biological structures could be printed. In a following study, the same group demonstrated printing jammed microgel bioinks within above-mentioned supramolecular hydrogel that enabled controlling cellular microenvironments via adjusting microgel properties.^104^

In another recent study, Luo *et al.* developed freeform reconfigurable embedded all-liquid (FREAL) bioprinting to print one liquid material within an immiscible liquid phase, in which the structural ink was stabilized by the noncovalent membrane at the interface.^106^ In a typical FREAL bioprinting, NIH 3T3-laden dextran/polyacrylamide was printed within PEO-diacrylate solution, in which the writing of the bioinks could be repeatedly revised and reconfigured, enabling adding, retracting, and modifying printed structures before the ink or the matrix is solidified. Complex 3D structures such as spring/tornado-shaped, barbell-shaped, double-spring/tornado-shaped, artery-like tree branches, and a goldfish skeleton structure were successfully printed using FREAL. FREAL was also combined with different nozzle designs, which further increased the complexity of printed structures. Inkjet bioprinting was also available for freeform bioprinting, where an alginate-based bioink was dispensed onto the liquid–air interface layer of a CaCl_2_ bath layer-by-layer.^28,45^ Christensen *et al.* used such inkjet printing system to bioprint vascular-like structures with both horizontal and vertical bifurcations with NIH 3T3-laden alginate.^28^ The high cell viability (>90%) indicated the feasibility of freeform inkjet printing of cellular structures.

The freeform bioprinting requires the use of a support bath with strict material properties as described above in detail. This also restricts the printable bioink formulations. Ji *et al.* developed an alternative bioprinting approach to create complex channels within bulk cell-laden hydrogels [Fig. 1(f)]. The supportive matrix, a photocurable hydrogel (e.g., methacrylated alginate or HA), was printed layer-by-layer during which each layer is exposed to blue light for a few seconds to create a partially crosslinked self-supporting layers. As the matrix was stacked, at a desired height, a sacrificial hydrogel pattern was printed within the top, uncrosslinked layer, followed by partial cross-linking, and matrix stacking steps continued until printing another pattern of sacrificial hydrogel or competition of the printing process. The construct was fully crosslinked at the end of the printing process using light, and the sacrificial hydrogel was washed away to form channels. The reported approach is a complementary technique to existing approaches to fabricate user defined and tunable channels, and does not require rapid fluid to solid transition of the support material (or shear-thinning behavior), which makes it more applicable for a wide range of commercially hydrogel systems.^92^

### Light-assisted bioprinting and biomaterial printing

**FIG. 8.**
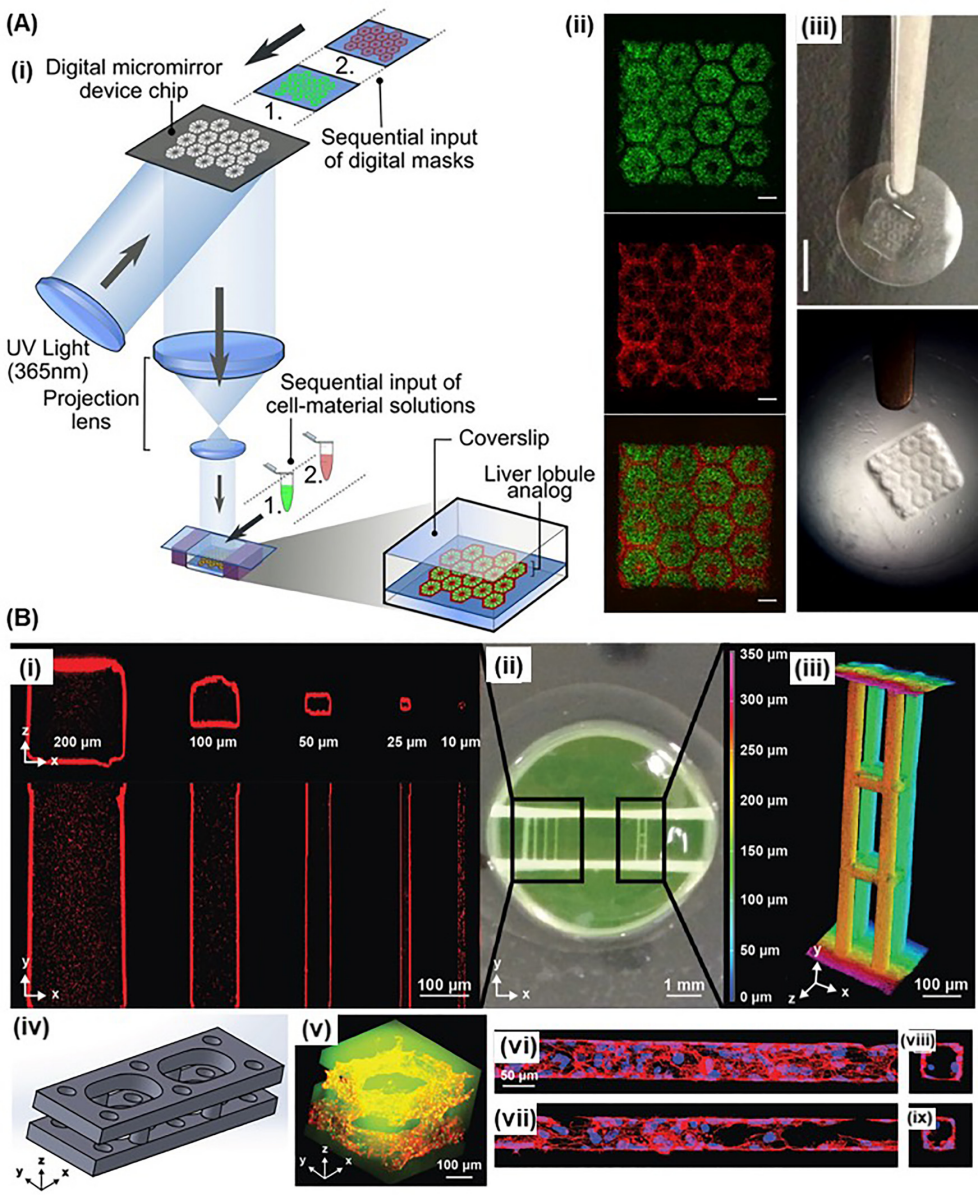
Light-assisted bioprinting and biomaterial printing: (a) complex patterned biomimetic human liver model using DMD-PP apparatus: (i) schematic illustration of a general printing process of the DMD-PP printing; (ii) fluorescent images of bioprinted hiPSC-HPCs (green) in 5% (wt./vol.) GelMA and supporting cells (red) (scale bars, 500 *μ*m); (iii) bioprinted 3D triculture construct. (Scale bar, 5 mm.).^113^ Adapted with permission from Ma *et al.*, “Deterministically patterned biomimetic human iPSC-derived hepatic model via rapid 3D bioprinting,” Proc. Natl. Acad. Sci. U.S. A. **113**(8), 2206–2211 (2016). Copyright 2016 Authors, licensed under a Creative Commons Attribution (CC BY) License. (b) Light-assisted bioprinting using user-programmable biomaterial photodegradation: (i) 3D printing of microvessels with micrometer-scale resolution; (ii) optical image of hydrogel with both parallel microchannels and 3D multilayered channel sets; (iii) a 3D image of photodegraded channels; (iv) a CAD design of the patterned channels; (v) 3D endothelialized channels generated within photodegradable fluorescent gels (green) (nuclei in green, F-actin in red); (vi)–(ix) longitudinal and cross-sectional view of the obtained endothelialization of the channels. Reprinted with permission from DeForest *et al.*, “Multicellular vascularized engineered tissues through user‐programmable biomaterial photodegradation,” Adv. Mater. **29**, ▪ (2017). Copyright 2017 John Wiley and Sons.

Vat photopolymerization-based printing approaches have gained recent attention due to their ability to create support-free complex structures and omnidirectional printing [Fig. 1(g)]. In particular, light-assisted printing using projection, including digital light processing (DLP) and continuous digital light processing (cDLP), has attracted more interest due to enhanced print speed as compared to SLA.^31,32,107–119^ For instance, Wang *et al.* developed a DLP system that projected computed visible light (514 nm) profile to cross-link fibroblast-laden GelMA/poly(ethylene glycol) diacrylate (PEGDA), which was initiated by Eosin Y-based photoinitiator.^32^ Considering the cytotoxicity of the UV that was pervasively used in vat polymerization-based bioprinting, the visible light projection not only printed highly vertical cell-laden structures with 50 *μ*m resolution, but also demonstrated 85% viability at least for 5 days. Although such vat polymerization-based 3D bioprinting is capable of printing freeform structures with high resolution, the printing process is normally restricted within the same vat of the photocurable resin, resulting in single-material printing. To address this issue, Choi *et al.* prepared multiple vats of different inks for multi-material SLA printing.^120,121^ Multi-material constructs were printed by dipping the printing platform into different vats prior to cross-linking. The printed construct was cleaned to remove the uncrosslinked ink before each time the platform was dipped into a different vat. This led to extra processing time to complete the print job. In another study, Chan *et al.* sequentially fed different inks/bioinks in a layer-by-layer manner, which demonstrated another solution to multimaterial SLA/DLP bioprinting.^122^ Using similar strategy, Han *et al.* developed a customized DLP approach, digital micromirror device projection printing (DMD-PP),^107^ in which poly(ethylene glycol) diacrylate (PEGDA) monomers were fed through a servo in a layer-by-layer manner for cross-linking, rather than cross-linking within a vat. This approach enabled feeding different inks at different layers, leading to multi-material complex printing. Using the same apparatus, Ma *et al.* printed triculture platforms that embedded hiPSC-derived hepatic progenitor cells (hiPSC-HPCs), HUVECs, and adipose-derived stem cells in GelMA constructs with hexagonal patterns [Fig. 8(a)].^113^ Compared with the 2D monolayer culture and 3D HPC-only model, the bioprinted triculture system showed enhanced expression of liver specific genes and key enzymes (such as cytochrome p450 enzymes).

In a recent study, Grigoryan *et al.* developed a cDLP system, which used synthetic and natural food dyes (e.g., tartrazine, curcumin, and anthocyanin), as well as gold nanoparticles as biocompatible photoabsorber recipes to formulate photocurable bioinks.^31^ In addition to commonly used PEGDA or GelMA, the couple of PEG-norbornene and PEG-dithiol were engineered for click-chemistry polymerization to reduce oxygen induced quenching and to enable tunable and faster cross-linking reaction. Using this bioink system, multivascular networks, functional intravascular topologies, and vascularized cell-laden liver tissue implants were fabricated. Moreover, the authors printed alveolus models with air-supplied air way atrium and the surrounding complex vascular network. While tidal ventilation and simultaneous perfusing of red blood cells were performed, the red blood cells were oxygenated by the ventilation.

In addition, multi-photon assisted manufacturing is also available for 3D complex bioprinting on a microscopic scale or even nano-scale.^36^ Two-photon polymerization (TPP) uses a near-infrared femtosecond laser which can be focused on a region to induce polymerization or cross-linking within that region.^36,123–127^ For instance, Xing *et al.* synthesized a high-efficient water-soluble TPP initiator, which enabled printing 3D adenovirus models with PEGDA hydrogels at a resolution of 92 nm.^127^ On the other hand, multi-photon-assisted photodegradation is also available for complex scaffold microfabrication.^128–131^ For instance, Arakawa and co-workers utilized a two-photon microscope to conduct precise molecular photolysis to fabricate complex 3D stromal cell-laden photodegradable PEG-based hydrogel constructs with microchannels, followed by endothelialization of the channels using HUVECs [Fig. 8(b)].^131^ The size of fabricated microchannels ranged from 10 *μ*m to 200 *μ*m, mimicking the native human capillary network.

## EMERGING APPROACHES

### Novel omnidirectional bioprinting approaches

As the advancements of 3D printing technology contributed to the recent advances of bioprinting, the emerging of novel 3D printing techniques will still be one of the major boosters of bioprinting in the future. Since early 1980s, when the first 3D printer was invented,^132^ the layer-by-layer manner is an intrinsic characteristic of this technology, no matter which type of 3D printing technique is used. The layers are always added in the direction of gravity, making conventional 3D printing techniques hard to print overhanging or bridging structures without support. This difficulty may be solved by adding new material in the normal direction to the substrate surface. Such considerations have encouraged researchers to invent omnidirectional printing techniques. Recently, some extrusion-based printers with flexibilities of 4 or more axis have been released, but such 3D printers are yet to be compatible with bioprinting.^133–135^ New ideas and conceptions have been raised, such as levitating 3D printing.

The conception of levitating additive manufacturing has been discussed and investigated in recent years.^6,10,136–139^ In 2016, The Boeing Company filed a patent for a magnetic levitating 3D printing technique.^140^ The printed object was levitated by magnetic force to balance the gravity, and more components were added to the initial object by extruding, propelling, or jetting. The additive components and the original part were cured by different stimuli, depending on the material used. Although the patented technique was proposed for metal or polymers, the magnified biological substance should be potentially compatible with such magnetic levitation systems.^6^ To enable magnetic levitation, Parfenov and co-workers added gadolinium (Gd^3+^) in media to culture chondrocytes. The magnetic chondrocytes were processed into spheroids and then assembled in a permanent magnetic field. Although the technique was only for spheroid assembly, it showed the potential of magnetic levitation for rapid 3D biofabrication.^141^ Moreover, acoustic levitation is also possible to be used to balance gravity, which does not require magnetic properties. Guo and co-workers developed 3D acoustic tweezers technology, which used surface acoustic waves to trap and place single cells or particles into 2D or 3D patterns.^142,143^ Although the bioprinted cellular architectures are not complex (and omnidirectionally printed), this technology demonstrated the unmatched feasibility to bioprint multicellular structures in a single-cell-scale.

### Volumetric bioprinting

**FIG. 9.**
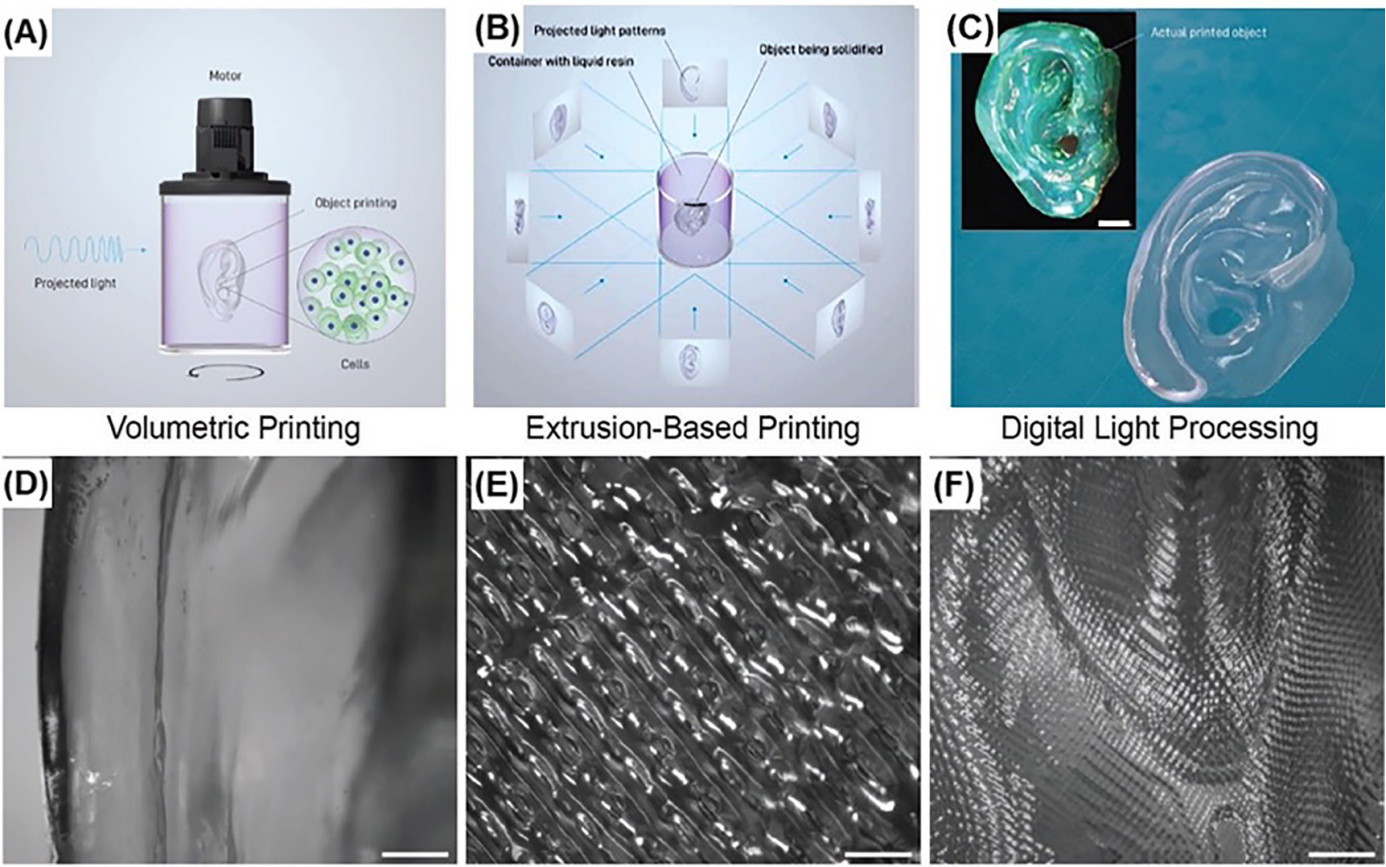
Overview of the volumetric bioprinting process: (a) the cell-laden gelRESIN reservoir connected to a rotating platform; (b) a schematic of tomographic projections used to print the human ear model; (c) stereomicrograph (inset, stained with with alcian blue) and optical image of the volumetric bioprinted ear model (printing time = 22.7 s; scale bar = 2 mm). (d)–(f) Close-up images of surface features for the comparison between volumetric, extrusion-based, and digital light processing. (scale bars = 500 *μ*m). Reprinted with permission from Levato *et al.*, “Volumetric bioprinting of complex living‐tissue constructs within seconds,” Adv. Mater. **31**, e1904209 (2019). Copyright 2019 John Wiley and Sons.

In 2019, Kelly and co-workers developed a volumetric additive manufacturing technique, called computed axial lithography (CAL).^144^ In this technique, light is projected onto a rotating vat, housing a photocurable resin. This allowed curing of the resin from all angles leading to fabrication of cm-scale constructs within minutes. Bernal and co-workers demonstrated successful application of CAL to bioprint meniscus-shaped articular implant using chondroprogenitor cell-laden GelMA-based (“gelRESIN”) bioinks.^145^ During the 28-day incubation, the bioprinted implant showed increased metabolic activity, ECM formation, and mechanical strength. Overall, CAL provided a new option for complex 3D bioprinting in a time- and cost-efficient manner, with ultrasmooth surface features (Fig. 9).

### Microfluidics-assisted bioprinting

**FIG. 10.**
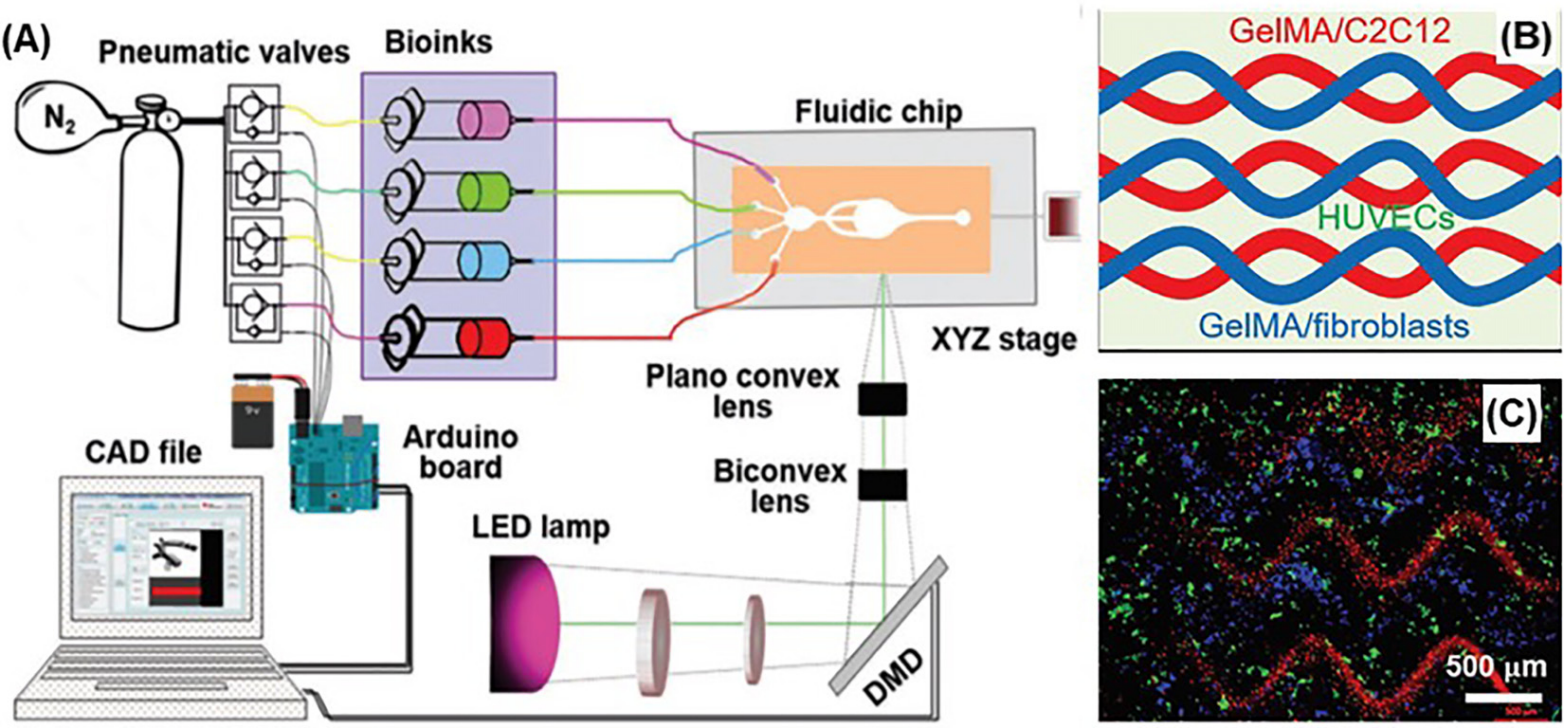
Microfluidics-enabled multimaterial maskless stereolithographic bioprinting: (a) Overview of the setup of the bioprinter, including a UV lamp (385 nm), optical lenses and objectives, a DMD chip, and a microfluidic device; (b) schematic of the design for printing skeletal muscle tissue; (c) bioprinted structure of GelMA containing patterned C2C12 cells (red) and fibroblasts (blue) after 48 h of culture. Reprinted with permission from Khademhosseini *et al.*, “Microfluidics‐enabled multimaterial maskless stereolithographic bioprinting,” Adv. Mater. **30**, e1800242 (2018). Copyright 2018 John Wiley and Sons.

There is a growing trend to integrate microfluidics technology with bioprinting (mainly extrusion-based bioprinting) for multi-material bioprinting to better mimic the ECM complexity of native tissues.^86,146–152^ Microfluidics-assisted bioprinting allows extrusion of multiple bioinks concurrently, sequentially, or as a mixture.^153^ For instance, Liu and co-workers developed a microfluidics integrated single printhead system with an ink pool of seven different ink formulations.^148^ Authors demonstrated perfusion and extrusion of a combination of multiple ink mixtures concurrently or a combination of distinct inks individually. A similar microfluidics-assisted extrusion-based bioprinting system was integrated with SLA bioprinting (Fig. 10)^146^ and with embedded freeform printing^150^ to fabricate multi-material 3D constructs. In a recent study, Skylar-Scott and co-workers developed a multi-material multi-nozzle 3D (MM3D) printing technique to print 3D objects, utilizing voxelated materials.^154^ Authors demonstrated fabrication of an origami pattern and a soft robot, utilizing multiple epoxy and silicone elastomer inks with varying stiffness. Although the MM3D technique was reported to print soft materials without cells, it showed a great potential as a high-throughput multi-material bioprinting approach.

### 4D bioprinting

**FIG. 11.**
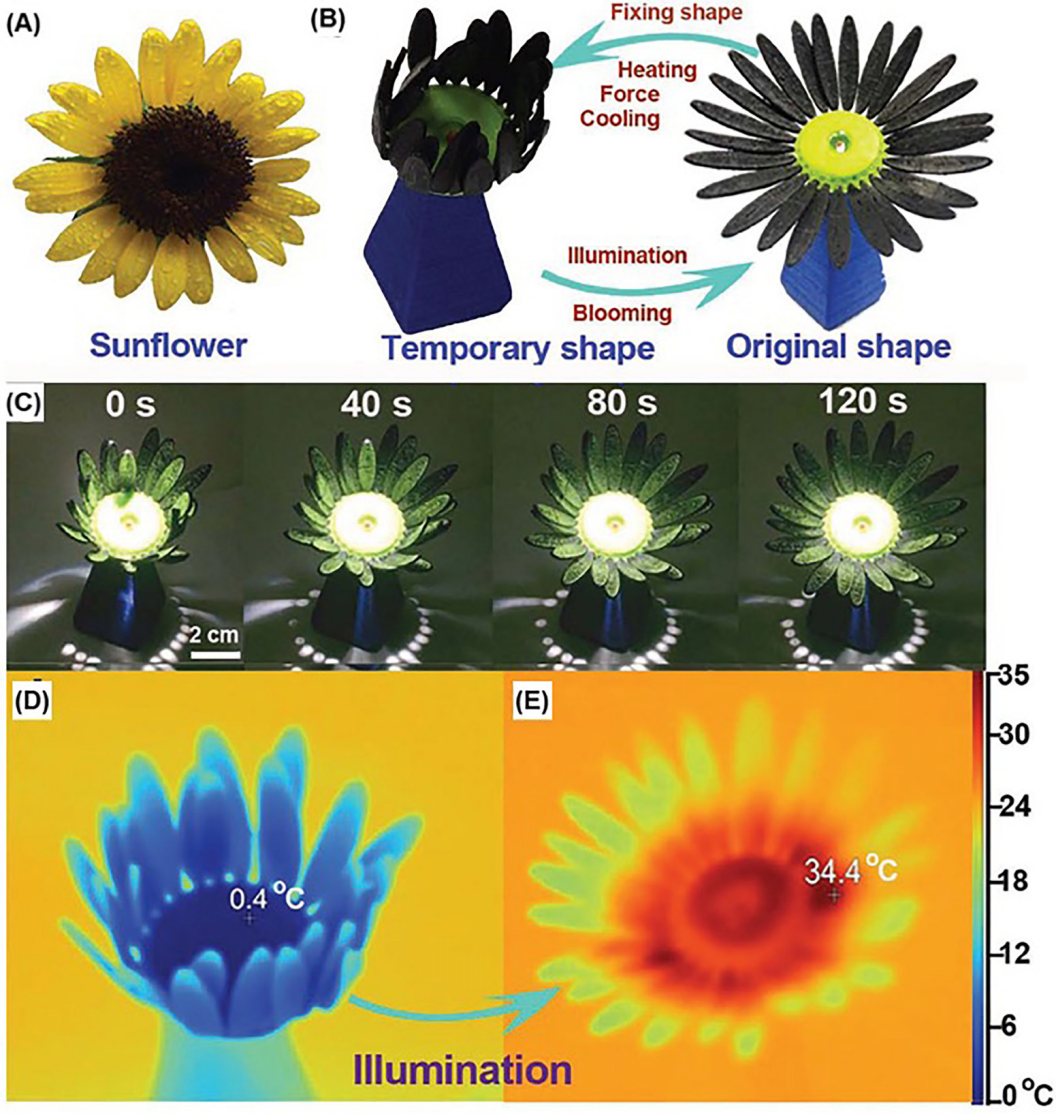
4D printing of light-responsive materials: (a) The real image of sunflower. (b) Light-responsive shape memory behavior of 3D printed sunflower from closed to opened state like the blooming of flowers. (c) The blooming process of 3D printed sunflower under illumination (light intensity: 87 mW cm^−2^). Images were obtained every 40 s. (d) and (e) The infrared image of sunflower before and after the illumination, displaying its temperature rose from 0.4 °C to 34.4 °C after 280 s illumination. Reprinted with permission from Chen *et al.*, “3D printed photoresponsive devices based on shape memory composites,” Adv. Mater. **29**, 1701627 (2017). Copyright 2017 John Wiley and Sons.

4D printing refers to 3D printing of stimuli responsive materials enabling change of the shape (the most commonly studied property) or other properties of the 3D printed construct in response to external stimuli.^155–160^ The external stimuli include physical [humidity,^161^ temperature,^162^ light (Fig. 11),^163,164^ electric field,^165^ or magnetic field^166^], chemical (pH^162,167^ or ion concentration^168^), and biological (enzyme^169,170^ or cellular traction^171^) origin. 4D printing is gaining recent attraction for biomedical applications,^160^ including stents,^172^ wound healing,^164,173^ drug delivery,^167^ and tissue constructs.^171,173^ Currently, 4D printed constructs enable cell-seeding post-printing when utilized for tissue engineering applications. However, majority of the currently utilized stimuli responsive materials are not suitable to be formulated as bioinks, and a few material platforms were formulated as bioinks.^160^ Considering that the cells can feel and interact with their microenvironment,^174–176^ there are great opportunities to utilize the stimuli responsive 4D printed constructs to dynamically regulate and program cell behavior and function.

### *In situ* bioprinting

**FIG. 12.**
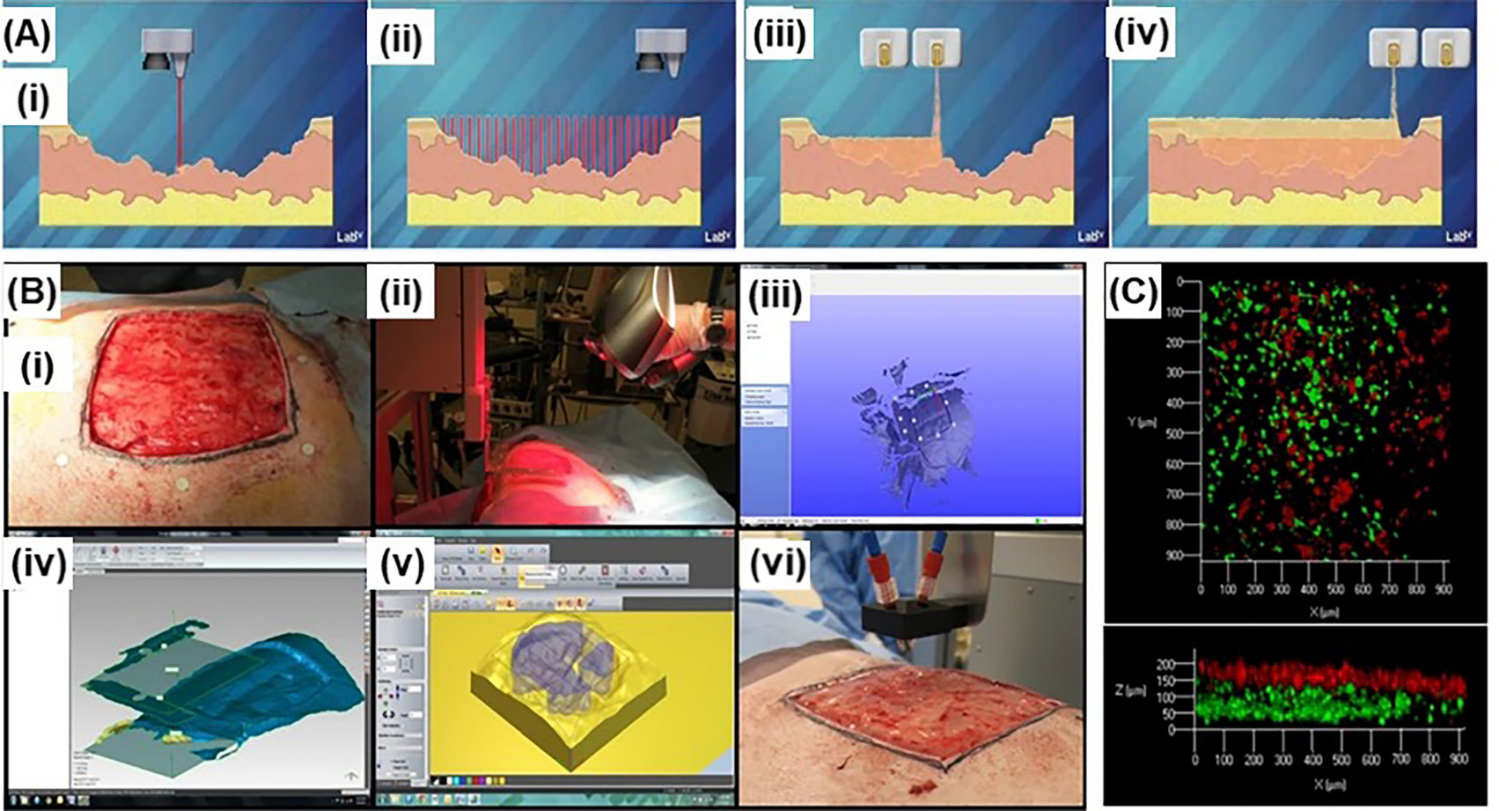
*In situ* bioprinting for skin wound healing: (a) Schematic illustration of *in situ* skin bioprinting: (i) wound scanning and information collection; (ii) designing printing process per collected information; (iii) and (iv) printing of proper bioinks at specific locations. (Images courtesy of LabTV—National Defense Education Program, Washington, D.C.). (b) Example of skin bioprinting process: (i) marking wound area as reference points; (ii) scanning the wound with a hand-held scanner; (iii) converting the data collected from the scanner into a standard coordinate system; (iv) computing the data and generating codes to direct printhead for printing; (v) and (vi) outputting the codes to the customized bioprinter and bioprinting. (c) A confocal microscopic image showing the layering of bioprinted fibroblasts (green) and keratinocytes (red). Adapted with permission from Albanna *et al.*, “In situ bioprinting of autologous skin cells accelerates wound healing of extensive excisional full-thickness wounds,” Sci. Rep. **9**(1), 1856 (2019). Copyright 2019 Authors, licensed under a Creative Commons Attribution (CC BY) License.

*In situ* bioprinting refers to bioprinting of living tissues directly onto the defect site in the living body. As compared to conventional bioprinting, where tissue is printed *in vitro* and usually matured in a bioreactor, living body acts as a bioreactor for *in situ* bioprinting.^177,178^ The feasibility of this technology has been shown for skin,^179–181^ cartilage,^182,183^ muscle,^184^ and bone tissue.^183,185,186^ A robotic arm or a handheld device is usually integrated with the *in situ* bioprinting systems to bioprint onto inherently uneven surfaces of the print site.^177,181,187^ Imaging is also important to detect the defect site to design an anatomically similar digital model for bioprinting. For instance, Albanna *et al.* developed a mobile extrusion-based *in situ* skin bioprinting system with integrated imaging technology for precise printing of dermal fibroblasts and epidermal keratinocytes directly into an injured site (Fig. 12).^180^ The skin bioprinter was able to replicate the layered skin structure. Layered autologous dermal fibroblasts and epidermal keratinocytes bioprinted with a fibrin/collagen hydrogel hydrogel carrier were shown to accelerate rapid wound closure and skin regeneration. Di Bella *et al.* developed a handheld *in situ* bioprinting device (biopen) and demonstrated the feasibility of coaxial extrusion of bioscaffold and cells into a cartilage defect in a sheep model.^188^ Authors reported favorable outcomes from early stages of cartilage regeneration at 8 weeks after *in situ* bioprinting. Instead of using depth sensors, McAlpine group developed *in situ* bioprinting approaches based on computer-aided vision systems.^181,187^ In 2018, Zhu *et al.* used tracking cameras to detect the fiducial markers on the target surfaces, which fed the pose information to the printer controller in real time to compute the toolpath of the printing, achieving *in situ* 3D printing of wearable devices or cell-laden hydrogels on moving freeform rigid surfaces.^181^ In the following study, the same group used an AI-powered 3D printing system to adapt the deformation and movement of the target surface, which enabled the *in situ* printing of stretchable strain sensors on breathing lungs with conductive hydrogels. Although the reported sensor fabrication was not a bioprinting process, this approach has great potential to be adapted for *in situ* bioprinting.^187^ Moreover, near-infrared light has been used for *in situ* bioprinting due to its enhanced penetration depth into skin.^189,190^ Chen *et al.* formulated GelMA-based bioinks supplemented with a nanoinitiator to cross-linking the GelMA under 980 nm near-infrared light.^190^ To demonstrate the *in vivo* bioprinting, a surgery was performed to create a subdermal muscle wound of a BALB/c mouse. Adipose-derived stem cell-laden bioink was injected under the skin to be crosslinked by the projected near-infrared light. Authors showed that the wound showed 80% closure within 10 days, whereas the untreated wound displayed 40% closure. Similarly, Urciuolo *et al.* demonstrated *in situ* bioprinting of 3D cell-laden hydrogels within or across tissues, including the dermis, skeletal muscle, and brain, of live mice.^189^ This approach relied on photocrosslinking of PEG-based bioinks under two-photon irradiation (700–950 nm).^189^ The use of multiphoton microscope ensured positional accuracy of the bioprinted structures *in situ*. When compared with the conventional open surgery approaches, *in vivo* bioprinting is minimally invasive and enable on-demand intervention with strong potential to present a new direction for bioprinting in clinic.

### Aspiration-assisted freeform bioprinting

**FIG. 13.**
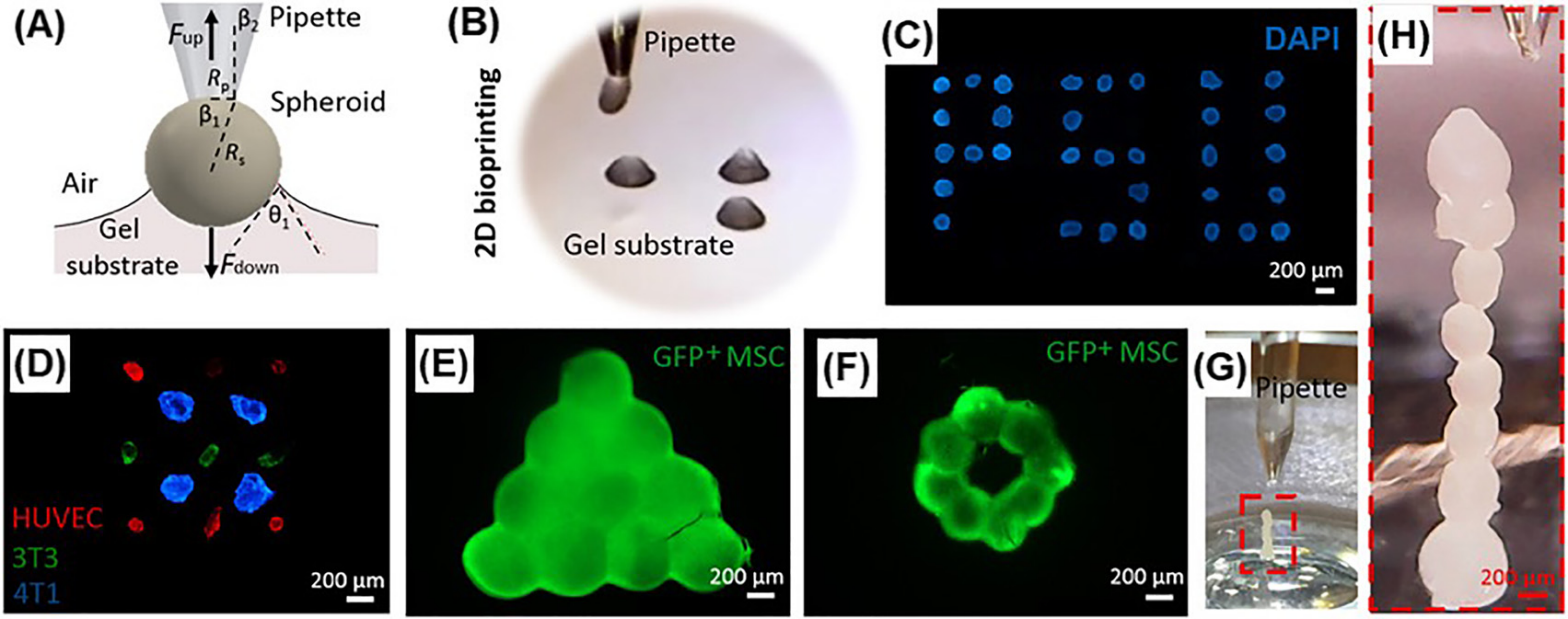
Aspiration-assisted bioprinting: (a) schematic illustration of critical parameters during bioprinting; (b) an image from the printer's traveling camera showing spheroid placement onto a gel substrate; (c)–(f) cellular patterns created by aspiration-assisted bioprinting cell spheroids; [(g) and (h)] stacking cell spheroids without supports. Adapted with permission from Ayan *et al.*, “Aspiration-assisted bioprinting for precise positioning of biologics,” Sci. Adv. **6**(10), eaaw5111 (2020). Copyright 2020 Authors, licensed under a Creative Commons Attribution (CC BY) License.

Aspiration-assisted bioprinting utilizes aspiration forces to pick, transfer, and precisely position tissue spheroids or strands, and relies on cell fusion to create the final tissue constructs (Fig. 13).^62,64,191–193^ It is possible to create scaffold free tissue constructs by stacking spheroids or strands, yet achievable complexity is limited due to issues to create self-supporting tissues without printing of support structures concurrently. Ayan *et al.* integrated aspiration-assisted bioprinting of spheroids with freeform bioprinting approach, developing aspiration-assisted freeform bioprinting.^194^ In this approach, tissue spheroids were precisely positioned into support bath composed of a self-healing yield-stress carbopol microgel system. Authors demonstrated feasibility of bioprinting circular cartilage tissues and triangle-shaped osteogenic tissues from spheroids. A similar approach is reported to bioprint spheroids within hydrogels with shear-thinning and rapid self-healing property.^195^ Cardiac microtissue models for disease modeling applications were fabricated using induced pluripotent stem cell-derived cardiac spheroids with spatially controlled cardiomyocyte and fibroblast cell ratios to replicate healthy and scarred cardiac tissue. Overall, these aspiration-assisted freeform bioprinting is an emerging technology that enables bioprinting of high-density tissues with precise control over microstructure and cellular heterogeneity.

## CONCLUDING REMARKS

This Review provides an in-depth summary of the 3D bioprinting approaches to develop complex and functional tissues and organs. Native tissues and organs are both anatomically and physiologically complex. 3D bioprinting approaches enabling precise positioning of multitude of bioinks, including a range of cells and ECM mimetic components, to form large-scale living tissues with embedded vasculature will help advance bioprinting field toward biomanufacturing of functional tissues and organs. In addition, it is crucial to develop optimized tissue maturation processes, including utilization of bioreactors or patient's body as a bioreactor, to achieve viable and functional tissues. 3D bioprinting evolved from limited shape and single material/cell printing approaches to multi-material omnidirectional bioprinting approaches leading to complex living tissues with built in vasculature. The emerging 4D bioprinting and *in situ* bioprinting technologies will further advance bioprinting complexity to develop stimuli responsive dynamic tissues and utilization of patient's own body as a bioreactor, respectively. The advancements of complex 3D bioprinting will only grow further in the future, which will enhance our ability to bioprint functional human-scale tissues and organs.

## AUTHORS' CONTRIBUTIONS

S.J. wrote the article and prepared the figures. M.G. outlined, wrote, and edited the article. All authors gave final approval for publication.

## Data Availability

Data sharing is not applicable to this article as no new data were created or analyzed in this study.
